# Quantum chemical study of molecular properties of small branched-chain amino acids in water

**DOI:** 10.1007/s00726-024-03437-y

**Published:** 2025-01-19

**Authors:** Roman Boča, Žofia Rádiková, Juraj Štofko, Beata Vranovičová, Cyril Rajnák

**Affiliations:** 1 Faculty of Health Sciences, University of SS Cyril and Methodius, 91701 Trnava, Slovakia; 2 Faculty of Natural Sciences, University of SS Cyril and Methodius, 91701 Trnava, Slovakia

**Keywords:** B3LYP, DLPNO-CCSD(T), 2-amino(iso)butyric acids, 3**-**amino(iso)butyric acids

## Abstract

Four aliphatic amino acids—α-aminobutyric acid (AABA), β-aminobutyric acid (BABA), α-aminoisobutyric acid (AAIBA) and β-aminoisobutyric acid (BAIBA) were investigated in water as a solvent by two quantum chemical methods. B3LYP hybrid version of DFT was used for geometry optimization and a full vibrational analysis of neutral molecules, their cations and anions in the canonical and zwitterionic forms (6 forms for each species). Ab initio DLPNO-CCSD(T) method was applied in the geometry pre-optimized by B3LYP. Calculated molecular descriptors involve dipole moment, quadrupole moment, dipole polarizability, energy of zero-point vibration and total entropic term which enter the standard Gibbs energy. In addition, a set of collective electronic and thermodynamic properties associated with redox process were evaluated: ionization energy, electron affinity, chemical hardness, molecular electronegativity, electrophilicity index, absolute oxidation and reduction potentials. A mutual comparison of these structural isomers including γ-aminobutyric acid (GABA) shows high degree of similarity in molecular descriptors. However, cluster analysis of 12 electro neutral, linear and branched amino acids with 2 – 6 carbon atoms discriminates them into five clusters. It is found that the electrophilicity index correlates with the absolute reduction potential along a straight line (24 items). The reduction potential for canonical structure varies between 1.21 V (glycine) and 1.45 V (AABA) whereas for the zwitterionic form it is visibly lower 0.52–1.11 V. The highest absolute reduction potential > 1.43 V is shown by α-amino acids: α-alanine, AABA (homoalanine) and AAIBA having 2-methyl or 2-ethyl functional group. The calculated absolute oxidation potential correlates with the adiabatic ionization energy and can be used as a criterion of the antioxidant capacity. According to thermodynamic data, the SPLET mechanism of the electron-proton coupled transfer is favored over the alternative SET-PT mechanism. This work contributes to the creation of a database of molecular properties of amino acids based on the same method and basis set.

**Supplementary Information:**

The online version contains supplementary material available at 10.1007/s00726-024-03437-y.

## Introduction

Amino acids represent a numerous class of organic compounds which play an important and specific role in life of plants and organisms. Some of them, mostly proteinogenic amino acids, were a subject of intense venture by experimental techniques side by side with theoretical (quantum chemical) methods. In order to follow up systematically, we studied two series of amino acids so far by a consistent methodology and the basis set: (i) linear-chain aliphatic amino acids (glycine, β-alanine, GABA, DAVA); (ii) branched-chain α-aliphatic amino acids (α-alanine, valine, lysine, isolysine). The present report is focused to four, less common isomers with the same molecular formula C_4_H_9_NO_2_: α-aminobutyric acid (AABA, homoalanine), β-aminobutyric acid (BABA), α-aminoisobutyric acid (AAIBA) and β-aminoisobutyric acid (BAIBA). A comparison with γ-aminobutyric acid (GABA) is also provided.

Structural formulas of the studies species are presented in Table 1. These refer to so called canonical structures possessing carboxyl –COOH and amino –NH_2_ groups. However, in water solutions the amino acids exist in the zwitterionic form having –COO^–^ and –NH_3_^+^ functional groups. Moreover, *N* rotatable C–C bonds bring a number of 3^*N*^ rotamers and the number of isomers is enriched by the spatial positions of hydrogen atoms.

**Table 1:**
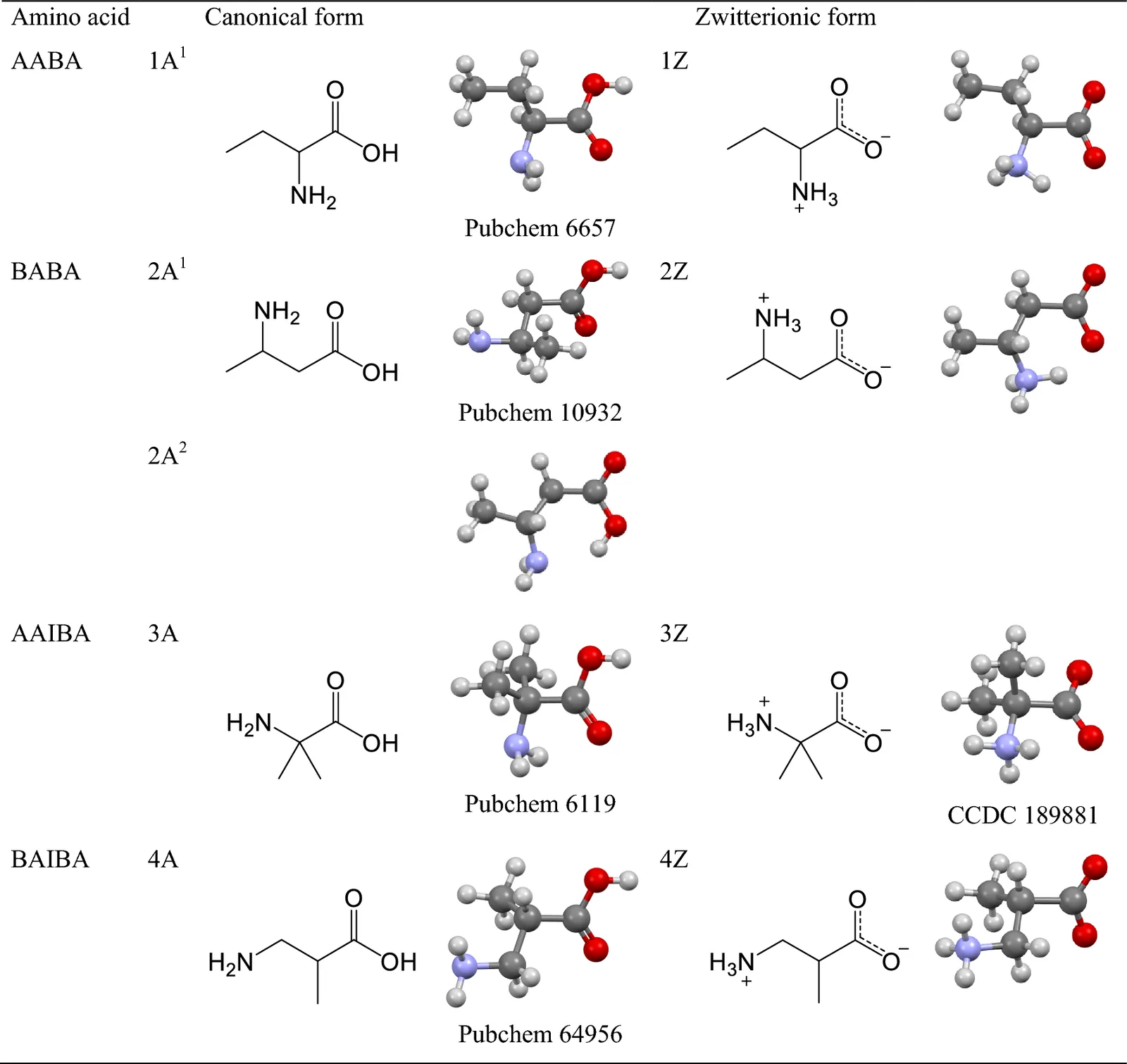
Structural formulas of linear and branched-chain aliphatic α-amino acids in their canonical (A) and zwitterionic (Z) forms

Color codes: C—black, H—white, N—blue, O—red

Physico-chemical properties of the studied amino acids are presented in Table 2. All of them are white solids with different solubility in water. The negative octanol/water partition coefficient points to a considerable hydrophobicity as log*P*_ow_ < 0. These species are non-essential and non-proteinogenic amino acids.

**Table 2:** Physico-chemical properties of five isomeric amino acids

Name	α-amino-butytic acid, homoalanine	β-amino-butytic acid	α-amino-isobutyric acid	β-amino-isobutyric acid	γ-amino-butyric acid
IUPAC name	2-amino-butanoic acid	3-amino-butanoic acid	2-aminoiso-butanoic acid	3-aminoiso-butanoic acid	4-amino-butanoic acid
Abbr	AABA	BABA	AAIBA	BAIBA	GABA
Molecular formula	C_4_H_9_NO_2_	C_4_H_9_NO_2_	C_4_H_9_NO_2_	C_4_H_9_NO_2_	C_4_H_9_NO_2_
Molar mass	103.12	103.12	103.12	103.12	103.12
Rotatable bonds	2	2	3	2	3
Consistency	White solid	Solid	Solid	Solid	White solid
Solubility in water	144 mg / cm^3^	1000 mg / cm^3^	181 mg / cm^3^		1300 mg / cm^3^
Partition coefficient log*P*_ow_	−3.17		−0.163 (est)	Very hydrophobic	−3.17
Acidity constants p*K*_a_	2.55 carboxyl 9.60 amino				4.02 carboxyl 10.56 amino
E-essential	No	No	No	No	No
PG-proteinogenic	No	No	No	No	No
Blood–brain barrier					No
PubChem CID	6657	10,932	6119	64,956	119
CCDC			189,881 (Z)		128,287 (Z)
DrugBank		–	DB02952		DB02530
HMBD	0000452	0031654	0001906	0003911	0000112
				0002166	
Refractivity /m^3^mol^−1 a^			21.21		25.46
Polarizability /Å^3 a^			10.33		10.62

Databases: PubChem, https://pubchem.ncbi.nlm.nih.gov/compound/name; Drugbank, https://go.drugbank.com/drugs/DBnumber; HMDB (Human Metabolome Database), https://hmdb.ca/metabolites/HMDBnumber; CCDC (Cambridge Crystallographic Data Center), https://www.ccdc.cam.ac.uk/structures/number

^a^Predicted data

There are about 500 amino acids (AA) found in nature, 22 of them are proteinogenic and only 20 are encoded by a standard genetic code. For humans, 9 of them are essential and must be taken with food; the rest of the proteinogenic AAs can by synthetized by humans themselves. Among the non-proteinogenic AA, our interest is focused on the isomers and enantiomers of aminobutyric acid (Vemula et al. 2017). These may exhibit identical chemical and physical properties, but different biological effects (Wang et al. 2020).

Alpha-aminobutyric acid (AABA) is a non-proteinogenic amino acid considered as a metabolite in AA synthesis or catabolism in humans (Effros 2011). It can be found in human kidney, liver tissue, serum, plasma, and cerebrospinal fluid as well (Lyssikatos et al. 2023a). Increased AABA levels were observed in liver diseases and alcoholism (Beyoğlu and Idle 2013) and in patients with atrial septal defect (Irino et al. 2016). In contrast, decreased levels were observed in elderly patients with depression (Adachi et al. 2019). Its association with physical activity and bone mineral density (Lyssikatos et al. 2023a) has been described as well. Due to its interference with glutathione homeostasis, it is assumed that AABA may have antioxidant and cardioprotective effects (Adachi et al. 2019). AABA was also proposed as a suitable indicator for liver damage (Rudnick et al. 2009), however this marker did not achieve universal acceptance and is not widely used (Ai et al. 2022). AABA is used in the pharmaceutical industry as a precursor for synthesis of anti-tuberculotic drug ethambutol and anti-epileptic drugs levetiracetam and brivaracetam (Xu et al. 2019). AABA can be used by nonribosomal peptide synthases. AABA is biosynthesized by transamination of oxobutyrate, a metabolite in isoleucine biosynthesis.

Beta-aminobutyric acid (BABA). BABA’s occurrence in nature is rare (Singh and Roychoudhury 2022). It was identified in cereals and cereal products, and cow milk. This AA can be found in plants, where it is responsible for inducing resistance to diseases, resilience to climate change and tolerance to biotic and abiotic stress (Singh and Roychoudhury 2022). Treatment with BABA leads to protection against several pathogens in plants (Zimmerli et al. 2000). In animals, BABA has been identified as a partial agonist of the inhibitory glycine receptor (Schmieden and Betz 1995). No relationship between BABA and human diseases could be identified so far (Wang et al. 2020). BABA is a secondary metabolite: non-essential metabolite that may serve a role as defense or signaling molecules.

Alpha-aminoisobutyric acid (AAIBA or AIB). Natural occurrence of this hydrophobic and in water insoluble molecule is very rare. However, AAIBA exists in all living organisms, ranging from bacteria to humans. AAIBA was found in some antibiotics produced by fungi (Matsuzaki et al. 1991). Labeled AAIBA was used experimentally to visualize malignant melanoma with positron emission tomography (Strauss and Conti 1991). In the past, the molecule was used in experiments to study the transport of ammino acids into various cells (Baran et al. 1972; Ronquist et al. 1976). In experimental conditions, its feature as a strong helical promotor was used to prepare synthetic polypeptides (Tsuchiya and Numata 2017) to increase peptides’ permeability into cells and its resistance to proteolytic enzymes (Taniguchi et al. 2019). Its use might be advantageous in developing new therapeutical strategies (Vemula et al. 2017).

Beta aminoisobutyric acid (BAIBA) is a very hydrophobic molecule, practically insoluble in water, and relatively neutral. BAIBA is a non-protein amino acid originating from the catabolism of thymine and valine. BAIBA can be biosynthesized from ureidoisobutyric acid; which is mediated by the enzyme beta-ureidopropionase. In humans, BAIBA is involved in the metabolic disorder called the beta-ureidopropionase deficiency pathway. In nature, BAIBA is found in two L- and D-enantiomeric forms (Tanianskii et al. 2019). D-BAIBA is produced from thymine, whereas L-BAIBA is a product of valine catabolism. BAIBA is secreted from muscle in response to physical activity and thought to induce browning of white adipose tissue by interfering with the PPARα mediated mechanism, to decrease weight gain, and to improve glucose tolerance (Roberts et al. 2014). This “myokine” plays probably a role in decreasing cardiometabolic risk by improving insulin sensitivity and reducing diet-related progression of obesity (Tanianskii et al. 2019). Although both enantiomers are produced by contracting skeletal muscle, it seems that D-BAIBA is associated with aging and physical fitness, whereas L-BAIBA is associated with bone mineral density in females (Lyssikatos et al. 2023b). L-BAIBA is assumed to decrease mitochondrial ROS production, support the function of osteocytes and to prevent bone loss (Hamrick and McGee-Lawrence 2018). Markedly decreased plasmatic concentrations of L-BAIBA are found in patients with beta-ureidopropionase deficiency and dihydropyrimidine dehydrogenase deficiency. These are autosomal recessive inborn errors of degradation of pyrimidine nucleotide bases thymine and uracil with neurological manifestation (Dobritzsch et al. 2022; Van Kuilenburg et al. 2004). On the other hand, increased concentrations of BAIBA were found in heart failure patients with Type 2 diabetes mellitus treated with sodium-glucose contransporter 2 inhibitors (Katano et al. 2022). Elevated excretion of BAIBA via urine was observed in lead poisoning (Farkas et al. 1987), cancer patients suffering from non-Hodgkin lymphoma and leukemia (van Gennip et al. 1987), and patients with ketoacidosis (Landaas and Solem 1983). BAIBA is potentially toxic.

## Methods of calculations

Methodology consistently resembles previous studies (Boča et al. 2024a, b) where it is specified in details. B3LYP variant of DFT has been used for the full geometry optimization of each species (neutral molecule, its cation and anion in UKS variant for open shells). This offers a set of electronic properties: ionization energy *E*_i_, electron affinity *E*_eg_, molecular electronegativity χ, chemical hardness η, electrophilicity index ω (Sen 1993; Pearson 1997; Parr et al. 1999). After the vibrational analysis, thermodynamic properties were evaluated: energy of zero-point vibration *E*_zpe_, internal energy *U*, entropy *S*^ø^, enthalpy *H*^ø^, Gibbs energy *G*^ø^, absolute oxidation *E*_ox_^ø^ and reduction potentials *E*_red_^ø^. The solvent effect was simulated using CPCM in water (Takano and Houk 2005). For geometry optimization, the B3LYP method with analytical gradients is suitable; it includes the electron correlation energy, but to an uncontrolled extent. The reliability of method is checked by comparing the calculated geometries with those contained in the CCDC structural data base.

The more sophisticated DLPNO-CCSD(T) method (Domain Localized Pair Natural Orbitals – Coupled Cluster Singles + Doubles + Triples) was also used; it is an ab initio post-Hartree–Fock method that includes almost the complete correlation energy. As a variant of CCSD(T), it is considered the gold standard (see Table S1 for comparison of methods). The energy data obtained with DLPNO-CCSD(T) are much more reliable compared to B3LYP as the correlation energy is included in a controlled way.

All calculations were done by the ORCA 5.0.4 package (Neese 2012, 2022; Neese et al. 2020). The molecular structures of the amino acids in their canonical forms A^1^ (often abbreviated as CF1) were retrieved from the database (PubChem 2004) and used as a starting point for full geometry optimization. For the zwitterions Z, the solid phase structures were found in the X-ray structural database (CCDC) (Lynch and McCleneghan 2002, Dobson and Gerkin 1996).

Huge data-set has been processed by advanced statistical methods to get latent correlations among parameters and objects: Cluster Analysis and Principal Component Analysis were exploited (Statgraphics 1982).

## Results and discussion

The results of the B3LYP calculations can be classified as follows: (i) optimized molecular geometry of the neutral and ionized species (*q* = + 1, 0, –1) for the canonical (A1) and zwitterionic forms, as shown in Tables S1 and S2; (ii) results of the vibrational analysis giving the total Gibbs energy *G*^ø,*q*^ for the above six forms of each species together with the total electronic energy *E*^*q*^; (iii) zero-point vibrational energy *E*_zpe_, lowest vibrational frequency ν_0_ and total entropic term *S*^ø^*T*^ø^; (iv) characteristics of a neutral molecule such as permanent dipole moment *p*, isotropic value of dipole polarizability *α*, isotropic value of quadrupole moment *Q*, solvated surface *S*, solvated volume *V*, HOMO and LUMO energies (highest occupied and lowest unoccupied molecular orbitals); (v) energy characteristics associated with redox processes, namely ionization energy *E*_i_, electron affinity *E*_g_, molecular electronegativity χ, chemical hardness η, electrophilicity index ω, absolute oxidation potential *E*_ox_^ø^ and absolute reduction potential *E*_red_^ø^. These outputs are listed in Table 3. Table S4 also contains results for eight other linear and branched amino acids obtained by the same methodology and basis set (Boča et al. 2024a, b).

**Table 3:** Molecular descriptors calculated by DFT-B3LYP method using adiabatic ionization/affinity processes in water for branched and linear amino acids

No	Molecule	Adiabatic redox properties /kcal mol^−1^										Properties of neutral molecules						
		*E*_i_	*E*_eg_	*χ*	*η*	*ω*	*E*_ox_^ø^	*E*_red_^ø^	*p*	*Q*	*α*	*S*	*V*	*E*_zpe_	*TS*^*ø*^	HO-MO	LU-MO	*ν*_0_
Canonical forms, A^1^																		
1A	AABA	142.3	−30.4	86.4	56.0	66.6	−6.13	1.45	1.927	−32.5	90.93	543	900	84.9	26.04	−161	−4.3	62
2A	BABA	138.4	−29.4	83.9	54.5	64.6	−5.92	1.42	3.116	−33.0	92.47	548	902	85.1	25.81	−157	−6.8	43
3A	AAIBA	142.4	−30.1	86.3	56.1	66.3	−6.11	1.43	2.076	−32.5	91.23	529	890	84.3	25.66	−163	−3.7	35
4A	BAIBA	138.1	−28.5	83.3	54.8	63.3	−5.92	1.37	2.457	−31.5	90.81	542	896	84.9	26.21	−156	−4.5	36
Zwitterionic forms, Z																		
1Z	AABA	148.6	−18.3	83.5	65.1	53.5	−6.42	1.00	13.62	−34.0	91.89	533	893	86.0	25.69	−158	1.1	41
2Z	BABA	144.3	−16.0	80.1	64.1	50.1	−6.22	0.90	15.13	−34.1	92.53	528	882	85.9	25.07	−155	1.8	62
3Z	AAIBA	147.5	−16.7	82.1	65.4	51.5	−6.38	0.91	13.62	−32.6	91.95	529	899	85.3	25.53	−157	0.4	70
4Z	BAIBA	138.3	−19.9	79.1	59.2	52.8	−5.94	1.12	21.45	−30.2	93.30	540	897	86.7	25.75	−148	−1.9	60
Abbreviation		I	A	X	H	O	Eo	Er	p	Q	al	S	V	Z	ST	Ho	Lu	n0

Basis set: def2-TZVPD (Weigend and Ahlrichs 2005). Energy quantities are in kcal mol^−1^; conversion factors: 1 kcal mol^−1^ = 4.184 kJ mol^−1^, 1 hartree = 627.5095 kcal mol^−1^; 1 eV = 23.06054 kcal mol^−1^. Standard temperature *T*^ø^ = 298.15 K. Absolute oxidation potential *E*_ox_^ø^ / V and absolute reduction potential *E*_red_^ø^ / V. Dipole moment *p* / D (*debye*, D = 3.336 × 10^–30^ A m s); isotropic quadrupole moment *Q* / e*a*_0_^2^, isotropic dipole polarizability *α* / *a*_0_^3^, solvated surface area *S* / *a*_0_^2^, solvated volume *V* / *a*_0_^3^ (*bohr*, *a*_0_ = 5.292 × 10^−11^ m); zero-point energy *E*_zpe_, lowest vibrational frequency ν_0_ / cm^−1^, total entropic term *S*^ø^*T*^ø^ in kcal mol^−1^. Adiabatic ionization energy *E*_i_ = *E*^+^ – *E*^0^, electron affinity *E*_eg_ = *E*^−^ − *E*^0^, (Mulliken’s) electronegativity *χ* = (*E*_i_ − *E*_eg_)/2, chemical (Pearson’s) hardness *η* = (*E*_i_ + *E*_eg_)/2 and (Parr’s) electrophilicity index *ω* = *χ*^2^/2*η*, the absolute redox potential *E*_abs_^ø^(L^0^/L^*q*^) [V] = − Δ_react_*G*^ø^[J mol^−1^]/*F*, Faraday constant *F* = 96,485 A s mol^−1^

Inspection of Table 3 confirms that the four branched isomers of GABA have nearly identical molecular properties. The exception is the dipole moment, which depends on the spatial separation of the barycenters of positive and negative charges (*p* = 1.93–3.12 debye). Adiabatic ionization energies for α-amino acids (AABA and AAIBA) are slightly higher compared to β-amino acids (BABA and BAIBA): *E*_i_ = 142 vs. 138 kcal mol^−1^. This is reflected in the absolute oxidation potential: *E*_ox_^ø^ = −6.1 vs. −5.9 V. Molecular electronegativity represents the electronic gradient – the driving force of electron transfer: χ = 83–86 kcal mol^−1^; chemical hardness refers to the electronic force constant – resistance to changes in electron density: η = 54–56 kcal mol^−1^; electrophilicity index – the electrophilic power ranges *ω* = 63–67 kcal mol^−1^. These data are close to other small amino acids (see Table S4). Zwitterionic forms have higher ionization energies (138–149), lower electron affinities in absolute value (−16 to −20), lower electronegativity (79–83), higher hardness (59–65), lower electrophilicity (50–53), a more negative oxidation potential (−5.9 to −6.4 V) and a less positive reduction potential (0.9–1.1 V); energy quantities are in kcal mol^−1^.

Based on the structural formula, the following coding was determined for individual amino acids: {number of carbon atoms, position of the amino group, position of methyl (m) or ethyl (e) groups} – Table 4. It is obvious that α-amino acids have higher adiabatic ionization energies (147–140) as β-amino acids (139–138) followed by linear GABA and DAVA (137, 136); data for A-forms in water. The trend for Z-forms is analogous to A-forms.

**Table 4:** Structural codes for studied amino acids in their canonical (A) forms, and calculated adiabatic ionization energies and electron affinities in water by B3LYP method

No	Amino acid	Type	Code	Formula	*E*_i_/kcal mol^−1^		*E*_eg_/kcal mol^−1^	
					A-form	Z-form	A-form	Z-form
5	Glycine	α	2–2		147.1	149.6	−25.1	−18.9
9	α-alanine	α	3–2-2m		142.7	148.5	−29.9	−18.8
3	AAIBA	α	4–2-2m-2m		142.4	147.5	−30.1	−16.7
1	AABA	α	4–2-2e		142.3	148.6	−30.4	−18.3
11	Leucine	α	6–2-4m-4m		141.2	148.7	−27.4	−18.5
12	Isoleucine	α	6–2-3m-3e		140.5	148.5	−29.5	−18.9
10	Valine	α	5–2-3m-3m		139.6	148.0	−29.6	−18.4
6	β-alanine	β, L	3–3		138.8	139.0	−29.7	−18.8
2	BABA	β	4–3-3m		138.4	144.3	−29.4	−16.0
4	BAIBA	β	4–3-2m		138.1	138.3	−28.5	−19.9
7	GABA	γ, L	4–4		137.5	135.0	−29.1	−11.9
8	DAVA	δ, L	5–5		135.9	133.3	−28.9	−11.4

Sorted according to the decreased B3LYP ionization energies for A-forms

Gas-phase Ultra-Violet Photoelectron Spectroscopy shows that the electron withdrawal occurs from the lone-pair of the nitrogen atom (Campbell et al. 1992; Cannington and Ham 1983). This is also confirmed by B3LYP calculations for glycine: the HOMO is mostly localized on the nitrogen atom (see Fig. S1). Therefore, the electron donating effect of methyl and/or ethyl substituents, as neighbors of the amino group, causes a decrease in the ionization energy.

**Fig. 1:**
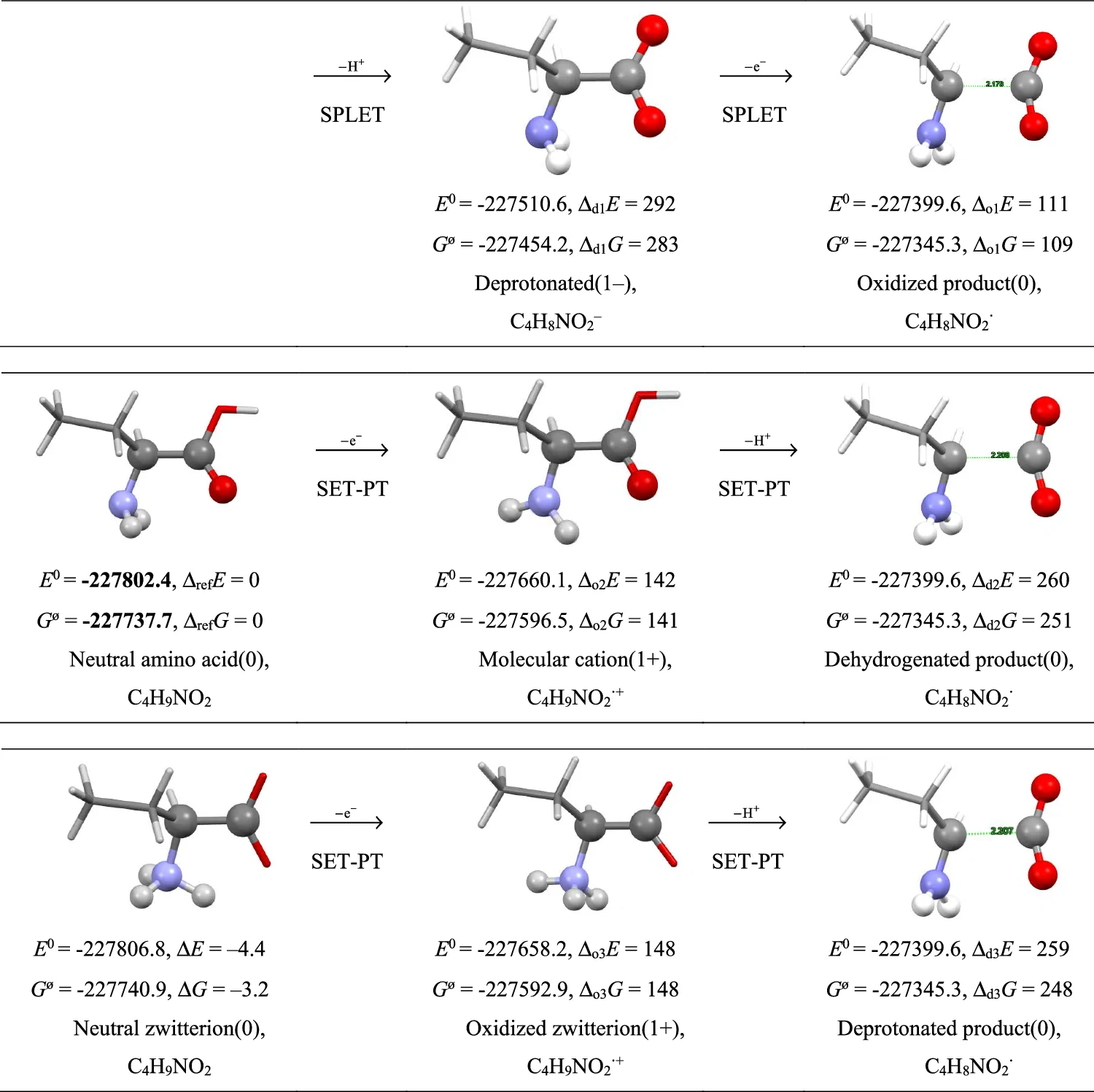
Results of geometry optimization by B3LYP for AABA, its oxidation and dehydrogenated products in water. Energies in kcal mol^−1^

Lower ionization energy is believed to increase the antioxidant capacity of the molecule (Dimič et al. 2017). The SET-PT mechanism (Single Electron Transfer followed by Proton Transfer) assumes the quenching of the free radical R^**·**^ by the sequence AA(OH) + R^**·**^ → R^–^ + AA(OH)^**·+**^ → RH + AA(O)^**·**^; the SPLET mechanism (Sequential Proton Loss Electron Transfer) assumes deprotonation as the first step AA(OH) → AAO^–^ + H^+^ and then AAO^–^ + R^**·**^ → AAO^**·**^** + **R^–^; the HAT mechanism (Hydrogen Atom Transfer) assumes a one-step process AA(OH) + R^**·**^ → RH + AA(O)^**·**^; the PCET (Proton Coupled Electron Transfer) involves two different routes for electron and proton transfer. All these mechanisms end in the same product – the amino acid residue AA(O)^**·**^ with the hydrogen atom removed. Attempts to optimize the geometry of such products have resulted in unstable species that spontaneously release CO_2_. Figure 1 shows the alternative electron-proton transfer (SET-PT) and/or proton-electron transfer (SPLET) pathways of AABA, both ending in the same unstable radical.

The SPLET mechanism requires Δ_d1_*G* = 283 kcal mol^−1^ for deprotonation as the first step, which needs to be corrected for the Gibbs energy of the solvated proton, i.e. Δ_d1_*G*_corr_ = Δ_d1_*G* + *G*^ø^(H^+^)_aq_ with *G*^ø^(H^+^)_aq_ = −262 kcal mol^−1^ (Zhan and Dixon 2001) with a yield of Δ_d1_*G*_corr_ = 21 kcal mol^−1^. Therefore, this step is less energetically demanding compared to the subsequent oxidation Δ_o1_*G* = 109 kcal mol^−1^. The concurrent SET-PT mechanism requires Δ_o2_*G* = 141 kcal mol^−1^ for oxidation as the first step, and then only Δ_d2_*G*_corr_ = Δ_d2_*G* + *G*^ø^(H^+^)_aq_ = 251–262 = −11 kcal mol^−1^ as the second step.

It must be mentioned that the kinetic mechanism requires identification of the transition state and evaluation of the transition state Gibbs energy Δ*G*^≠^ (Galano 2007; Leon-Carmona and Galano 2011; Galano and Martinez 2012).

The ionization energy is the main factor influencing the reaction Gibbs energy for oxidation and then it dominates the absolute oxidation potential

1 $$\begin{gathered} E_{{{\mathrm{ox}}}}^{\emptyset } = - G_{{{\mathrm{ox}}}}^{\emptyset } /F = - (G^{ + ,\emptyset } - G^{0,\emptyset } )/F \\ = - (U^{ + ,\emptyset } - U^{0,\emptyset } )/F + (TS^{ + ,\emptyset } - TS^{0,\emptyset } )/F \\ \doteq - \underbrace {{(E^{ + } - E^{0} )}}_{{E_{{\mathrm{i}}} }}/F - (E_{{{\mathrm{zpe}}}}^{ + } - E_{{{\mathrm{zpe}}}}^{0} )/F \\ \end{gathered}$$

**Fig. 2:**
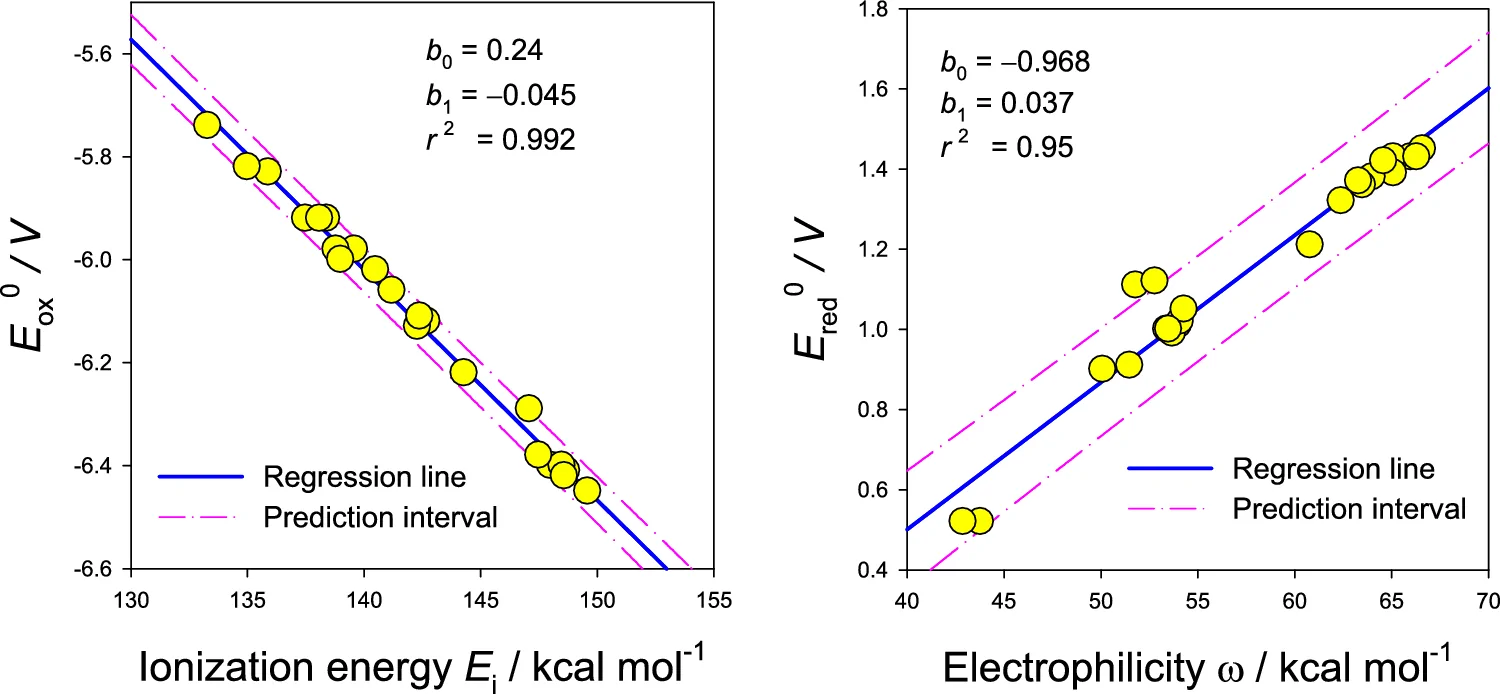
A correlation of the absolute oxidation potential with ionization energy and absolute reduction potential with the electrophilicity index (24 entries for canonical and zwitterionic forms of 12 amino acids, B3LYP data). Regression line: *y* = *b*_0_ + *b*_1_*x*

Here, the entropic terms *TS* terms and the zero-point vibration terms *E*_zpe_ almost cancel, and the correction to enthalpy cancels exactly. The correlation of the absolute oxidation potential with the adiabatic ionization energy is shown in Fig. 2: the correlation is almost perfect (correlation coefficient *r*^2^ = 0.99). This suggests that the tedious vibrational analysis can eventually be skipped for reliable estimation of the absolute oxidation potential; however, geometry optimization for the neutral and ionized molecules needs to be completed.

**Fig. 3:**
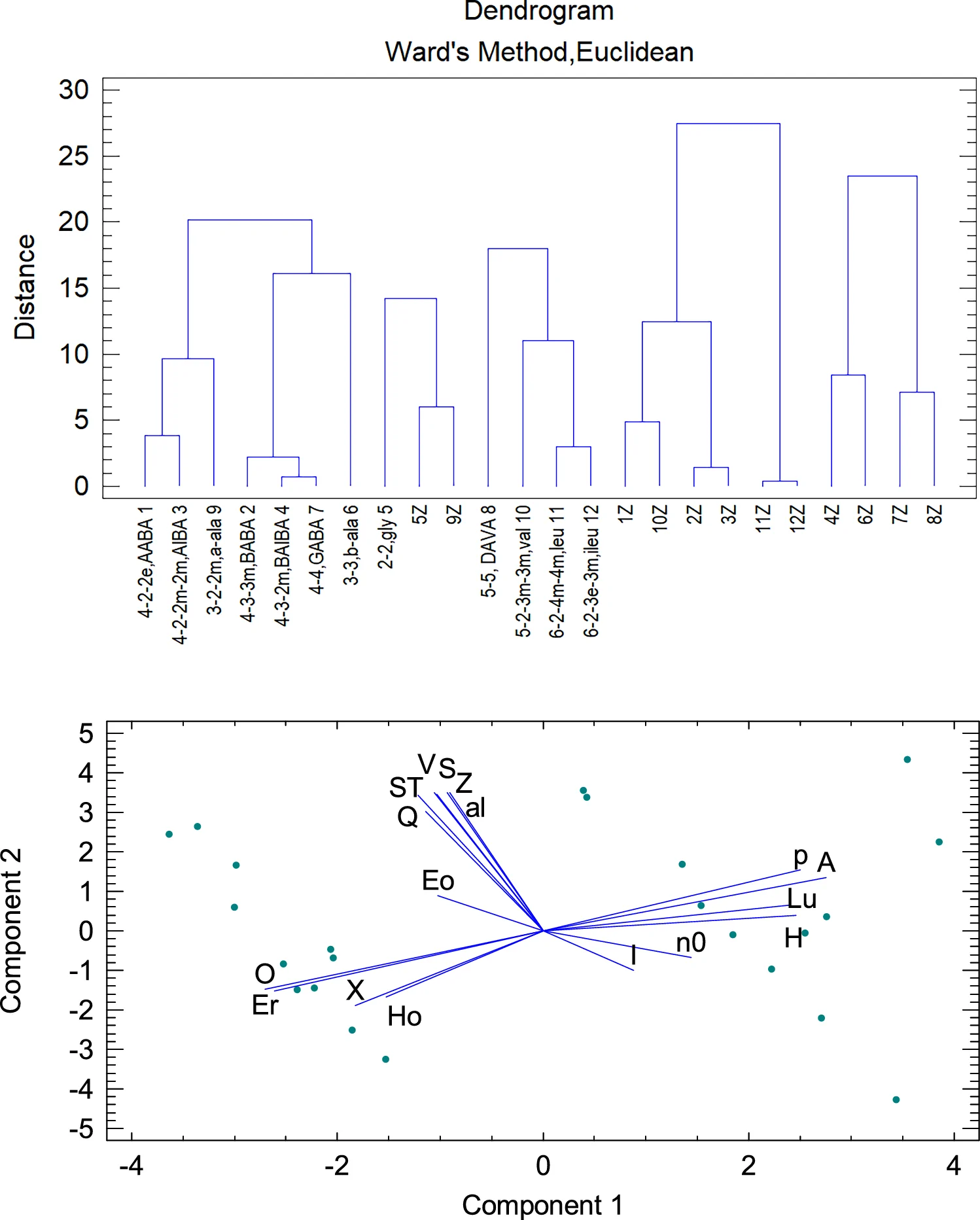
Classification of branched and linear amino acids by statistical methods (24 entries): CA (Ward’s method, metric – Euclidean) and PCA (bottom panel). I—ionization energy, A—electron affinity, X—electronegativity, H—hardness, O—electrophilicity index, p—dipole moment, Q—absolute value of the quadrupole moment, al—polarizability, Eo—absolute oxidation potential, Er—absolute reduction potential, Z—zero-point vibration, n0—lowest vibrational frequency, S—solvated surface, V—solvated volume, Ho—absolute value of the HOMO energy, Lu—energy of LUMO

The correlation of the absolute reduction potential *E*_r_^ø^ with the electrophilicity index *ω* is also shown in Fig. 3. The electrophilicity index is expressed as *ω* = (1/4)(*E*_i_ − *E*_eg_)^2^/(*E*_i_ + *E*_eg_) and includes both, ionization energy and electron affinity. The reduction potential *E*_red_^ø^ = −Δ_red_*G*^ø^/*F* ~ −*E*_eg_/*F* is dominated by electron affinity. There is no rational relationship between these quantities but there is an empirical correlation confirmed by statistical methods. The reduction potential could find a relationship with the anti-reduction capacity; however, experimental data are lacking.

According to Table S4, the reduction potential for the canonical structure ranges between 1.21 (glycine) and 1.45 V (AABA), while it is noticeably lower for the zwitterionic form: 0.52–1.11 V. The maximum absolute reduction potential > 1.43 V exhibit α-amino acids {α-alanine, AABA, AAIBA} with 2-methyl or 2-ethyl functional groups: 3–**2-2m**, 4–**2-2e** and 4–**2-2m**-2m. These compounds are good candidates for significant oxidation (antireduction) activity. Close to those values are {valine, *iso*-leucine, BABA} with codes 5–**2-3m**-3m, 6–**2-3e**-3m and 4–**3-3m**.

Table S4, which contains data for 12 amino acids, has 17 × 12 = 204 entries for canonical forms and the same number for zwitterionic forms. Such a huge data set was processed by advanced statistical methods. This dataset could be expanded to include other aliphatic and aromatic amino acids in the future. Statistical methods can help understand broader trends in the behavior of other amino acids or small molecules.

Cluster Analysis (CA, Ward's method) shows that the entire data set containing 24 objects is divided into 5 clusters according to their similarity expressed by the “distance” defined through the Euclidean metric (Fig. 3).

1. The four studied complexes **1** – **4** span the same similarity cluster along with other three-C and four-C species.
2. This cluster is divided into a subgroup formed by small α-amino acids {AABA (abbr. 4–**2-2e**), AAIBA (4–**2-2m-2m**), α-alanine (3–**2-3m**)}.
3. The second subset contains small β-amino acids {BABA (abbr. 4–**3-3m**), BAIBA (4–**3-2m**), β-alanine (3–3) and, in addition, GABA (4–4)}.
4. Another cluster is formed by more extended amino acids containing five or six carbon atoms: {DAVA (5–5), valine (5–**2-3m-3m**), leucine (6–**2-4m-4m**) and isoleucine (6–**2-3m-3e**)}.
5. Glycine (2–2) and its Z-form are classified in their own cluster.
6. Zwitterions cover the remaining clusters.

Principal component analysis (PCA) yields information which observables are correlated (adjacent rays), anticorrelated (opposite rays), or uncorrelated (normal to each other).

1. The best correlation shows the pair {O, Er}, which is the electrophilicity index and the absolute reduction potential. Note that the relationships Er ~ −Δ*G*_red_ ~ −A approximately hold, so electron affinity anticorrelates with absolute reduction potential.
2. The ionization energy {I, Eo} anticorrelates with the absolute oxidation potential, which originates in Eo ~ −Δ*G*_ox_ ~ −I relationships.
3. There is a set of bulk properties that depend essentially on molar mass: solvated surface and volume, dipole polarizability, zero-point vibration and total entropic terms. Therefore, these properties form their own cluster {S, V, al, Z, ST} which can be enriched with the absolute value of the quadrupole moment |Q|.
4. The dipole moment dos not correlate with other descriptors because it depends on the specific location of the center of negative and positive charge.

Supplementary information includes a matrix plot with all pair-wise graphs. They are quantified using the pairwise correlation coefficients; highest ρ > 0.95 display {*E*_i_, *E*_ox_} and {*E*_eg_, *E*_red_} pairs.

Quantum chemical calculations were repeated using the advanced DLPNO-CCSD(T) method, which explicitly includes the major part of the correlation energy (Table 5). Calculations were performed in a fixed geometry pre-optimized with B3LYP. This method offers essentially analogous results compared to B3LYP calculations. Slightly higher ionization energies *E*_i_ gave more negative oxidation potentials (−6.2 to −6.4 V) when estimated using *E*_ox_^*^ = −*E*_i_/*F*. For zwitterions, these values are even more negative (−6.4 to −6.8 V). The absolute reduction potentials are less positive (1.12–1.21 V) and (0.7–0.9 V), respectively. Electronegativity for zwitterions is lower compared to canonical structures, hardness higher (64–70) *vs* (59–60), and electrophilicity lower (53–55) *vs* (61–64); data in kcal mol^−1^.

**Table 5:** Molecular descriptors calculated by DLPNO-CCSD(T) method using adiabatic ionization/affinity processes in water

No	Molecule	Adiabatic redox properties							Properties of neutral molecules					
		*E*_i_	*E*_eg_	*χ*	*η*	*ω*	*E*_ox_^*^	*E*_red_^*^	*p*	*Q*	*S*	*V*	HOMO	LUMO
Canonical forms, A^1^														
1A	AABA	148.6	−27.8	88.2	60.4	64.4	−6.44	1.21	2.012	−32.3	543	900	−255	20.4
2A	BABA	144.4	−26.3	85.3	59.0	61.7	−6.26	1.14	3.338	−32.9	548	902	−249	20.4
3A	AAIBA	147.9	−27.9	87.9	60.0	64.4	−6.41	1.20	2.159	−32.4	529	891	−257	20.3
4A	BAIBA	143.9	−26.0	84.9	58.9	61.2	−6.24	1.12	2.628	−31.4	542	896	−249	20.1
Zwitterionic forms, Z														
1Z	AABA	157.2	−18.4	87.8	69.4	55.5	−6.82	0.80	14.20	−33.9	533	893	−259	20.2
2Z	BABA	152.9	−16.6	84.7	68.1	52.7	−6.63	0.72	15.68	−34.0	528	882	−255	20.5
3Z	AAIBA	156.4	−16.9	86.6	69.7	53.8	−6.78	0.73	14.18	−32.5	529	899	−255	20.2
4Z	BAIBA	147.9	−19.9	83.9	64.0	55.0	−6.41	0.86	21.9	−30.0	540	897	−247	19.0
Abbreviation		I	A	X	H	O	Eo	Er	p	Q	S	V	Ho	Lu

Basis set aug-cc-pVTZ (Kendall et al. 1992; Weigend et al. 2002). For units see Table 3

## Conclusions

Four small, less common, almost forgotten amino acids were investigated by two quantum chemical methods in water as a solvent. They are all branched-chain isomers of linear GABA with the molecular formula C_4_H_9_NO_2_. The main attention is paid for two classes of properties: (i) molecular geometry and electronic properties of neutral species in the canonical and zwitterionic forms (molecular surface, molecular volume, dipole moment, isotropic quadrupole moment, isotropic dipole polarizability, zero-point vibration energy, entropic term, lowest vibrational frequency); (ii) adiabatic redox properties (ionization energy, electron affinity, molecular electronegativity, chemical hardness, electrophilicity index, absolute oxidation and reduction potentials). It was confirmed that the zwitterionic forms are more stable compared to canonical structure in water by about 4 kcal mol^−1^ (taking onto account the difference in electronic energy and/or standard Gibbs energy); the exception is BAIBA for which the canonical structure is slightly more stable than the zwitterionic structure.

The new data for 4 amino acids were compared with data for 8 other amino acids calculated by the same methodology. Molecular descriptors (408 entries in total) were processed by advanced statistical methods. Cluster Analysis allowed the classification of 24 species into several clusters: small α-amino acids (AABA, AAIBA, α-alanine), small β-amino acids (BABA, BAIBA, β-alanine) and GABA, more widespread amino acid with 5 and 6 carbon atoms (DAVA, valine, leucine, isoleucine). Own cluster is formed of glycine in its canonical and zwitterionic forms. The rest of the zwitterions cover the remaining two clusters.

Principal Component Analysis brings information that the absolute reduction potential *E*_red_^ø^ correlates with the electrophilicity index ω, and the absolute oxidation potential *E*_ox_^ø^ is anticorrelated with the adiabatic ionization energy *E*_i_. Bulk molecular properties, such as molecular surface and volume, quadrupole moment, dipole polarizability, zero-point vibrational energy *E*_zpe_ and total entropic term *TS*^*ø*^, are correlated with each other.

A matrix plot of variable pairs visualizes which properties correlate along a smooth curve (line). This is quantified by calculating pair-wise correlation coefficients; the highest *ρ* > 0.95 show {*E*_i_, *E*_ox_} and {*E*_eg_, *E*_red_} pairs. The corresponding graphs confirm the linear behaviour *y* = *b*_0_ + *b*_1_*x*. This is an extraordinary result that shows that thermodynamic parameters, such are absolute oxidation and reduction potentials, can eventually be approximated using electronic parameters only, without tedious vibrational analysis.

The calculated absolute oxidation potential has been related to the antioxidant capacity of the amino acid AA(OH) when the SET-PT mechanism (Single Electron Transfer followed by Proton Transfer) is on. This mechanism, together with SPLET and HAT alternatives, ends with the same product – the amino acid residue AA(O)^**·**^ with the hydrogen atom removed. Attempts to optimize the geometry of such products have resulted in unstable species that spontaneously release CO_2_. Based on thermodynamic data, electron-proton coupled transfer favors SPLET mechanisms for AABA (with steps of 21 + 109 kcal mol^−1^) over the alternative SET-PT mechanism (with steps of 141 – 11 kcal mol^−1^).

## Supplementary Information

Below is the link to the electronic supplementary material.

Supplementary file1 (PDF 1184 KB)

## Data Availability

All computational protocols are available from the corresponding author on request.
