# Supplementary material for: Quantum chemical study of molecular properties of small branched-chain amino acids in water
Source: Amino Acids. 2025 Jan 19;57:11. doi: 10.1007/s00726-024-03437-y (PMC11743389; doi:10.1007/s00726-024-03437-y)
Supplement: Supplementary file 1 [file 726_2024_3437_MOESM1_ESM.pdf]

Quantum chemical study of molecular properties of small branched-chain amino acids in water

Roman Boča · Žofia Rádiková · Juraj Štofko · Beata Vranovičová · Cyril Rajnák

Supplementary information

Table S1. Calculated total energy and electron correlation increments (a.u., hartree) for AABA.

Method	E^0[SCF]	E[correlation]	%	E[total]	CPU ~ M [a]
B3LYP	-363.02016906		?		M^3
Hartree-Fock	**-361.06406**723		-		M^3
MP2	**-361.06406**723	**-1.50917**625	93.7	-362.57324348	M^4, M^5
DLPNO-CCSD	**-361.06406**253	**-1.54766**712	96.1	-362.61172966	M^5
CCSD	**-361.06406**723	**-1.54757**181	96.1	-362.61163904	M^6
DLPNO-CCSD(T)	**-361.06406**723	**-1.60588**965	99.7	-362.66995688	M^6
CCSD(T)	**-361.06406**254	**-1.60997**133	100	-362.67403387	M^7

[a] AABA: 16 atoms, M = 571 basis functions (orbitals) for aug-cc-pVTZ basis set. CPU depending on basis set size M. % - percentage of the correlation energy with respect to CCSD(T). Data generated by ORCA 6.0.0. 1 hartree = 627.5095 kcal mol^{-1}.

Table S2. Structure and molecular properties for various conformers of AABA.[a]

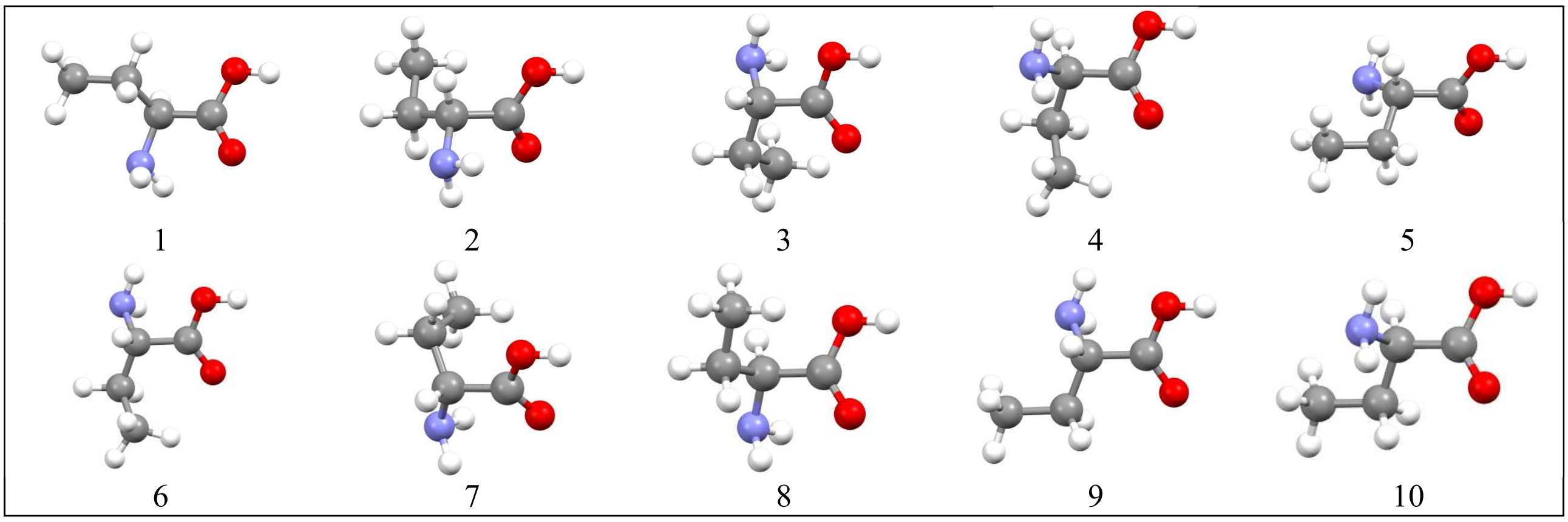

No	E^0	ΔE^0	$G^{\ominus}$	$\Delta G^{\ominus}$	p /debye	S /a_0^2	V /a_0^3	E_{zpe}	$TS^{\ominus}$	HOMO	LUMO
1	-227802.39	0	-227737.71	0	1.927	543	900	84.90	26.01	-161.14	-4.29
2	-227801.99	0.40	-227737.21	0.50	1.954	539	896	84.97	25.96	-161.69	-6.53
3	-227800.94	1.45	-227736.17	1.54	2.397	533	893	84.97	25.97	-156.81	-5.68
4	-227801.99	0.40	-227737.01	0.70	1.976	536	896	85.06	25.79	-162.57	-5.39
5	-227802.39	0.00	-227737.71	0.00	1.912	546	902	84.90	26.01	-161.04	-4.43
6	-227802.00	0.38	-227737.23	0.48	1.949	539	896	84.96	25.96	-161.62	-6.43
7	-227802.00	0.39	-227737.11	0.60	1.976	536	897	85.00	25.86	-162.58	-5.37
8	-227802.02	0.37	-227737.09	0.62	1.957	543	903	85.05	25.86	-161.71	-6.32
9	-227802.40	-0.01	-227737.66	0.05	1.920	546	902	84.93	25.97	-161.07	-4.41
10	-227802.41	-0.02	-227737.65	0.06	1.921	545	901	84.95	25.96	-161.10	-4.42

[a] All energies in kcal mol^{-1}. E^0 – total electronic energy, $G^{\ominus}$ – standard Gibbs energy, ΔE^0 and $\Delta G^{\ominus}$ energies relative to the first conformer, p – dipole moment, S – solvated surface, V – solvated volume, E_{zpe} – zero-point vibration energy, $TS^{\ominus}$ – total entropic term.

Table S3. Total electronic energies and standard Gibbs energies calculated by B3LYP and DLPNO-CCSD(T) methods in optimized structure of L^q.[a]

B3LYP /def2-TZVPD		Canonical forms A[1]			Zwitterionic forms Z			ΔE
Molecule/ion		A^+	A^0	A^-	Z^+	Z^0	Z^-	ΔG^o
1) AABA	E^q	-227660.07	-227802.39	-227832.78	-227658.20	**-227806.81**	-227825.15	–4.4
	$G^{o,q}$	-227596.49	-227737.74	-227771.12	-227592.91	-227740.94	-227764.09	–3.2
2) BABA	E^q	-227665.34	-227803.78	-227833.19	-227666.32	-227810.61	-227826.61	–6.8
	$G^{o,q}$	-227602.33	-227738.85	-227771.50	-227600.92	-227744.42	-227765.08	–5.6
3) AAIBA	E^q	-227660.77	-227803.15	-227833.30	-227660.33	-227807.82	-227824.51	–4.7
	$G^{o,q}$	-227597.89	-227738.75	-227771.79	-227595.13	-227742.37	-227763.25	–3.6
4) BAIBA	E^q	-227662.96	-227801.11	-227829.61	-227663.81	-227802.11	-227822.04	–1.0
	$G^{o,q}$	-227599.92	-227736.52	-227768.1	-227598.33	-227735.58	-227761.30	+0.9
		D^0	D^-					
1') AABA	E^q	-227399.6	-227510.61					
	$G^{o,q}$	-227345.3	-227454.24					

DLPNO-CCSD(T) /aug-cc-pVTZ		Canonical forms A[1]			Zwitterionic forms Z			ΔE
Molecule/ion		A^+	A^0	A^-	Z^+	Z^0	Z^-	
1) AABA	E^q	-227430.19	-227578.79	-227606.59	-227424.70	**-227581.88**	-227600.23	–3.1
2) BABA	E^q	-227435.64	-227580.08	-227606.47	-227432.79	-227585.70	-227602.34	–5.6
3) AAIBA	E^q	-227434.12	-227582.07	-227609.96	-227428.96	-227585.35	-227602.30	–3.3
4) BAIBA	E^q	-227433.88	-227577.85	-227603.85	-227429.85	-227577.74	-227597.62	+0.11

[a] All energies in kcal mol^{-1}; conversion: 1 hartree = 627.5095 kcal mol^{-1}; 1 kcal mol^{-1} = 4.184 kJ mol^{-1}. Stability $\Delta E = E^0(Z) - E^0(A)$; $\Delta G = G^0(Z) - G^0(A)$. More stable electroneutral isomer is **bold typed**. D – deprotonated species.

Table S4 Optimized geometries of amino acids (canonical forms A[1]) in water by B3LYP/def2-TZVPD.

A^1 form	L^+	L^0	L^-
AABA	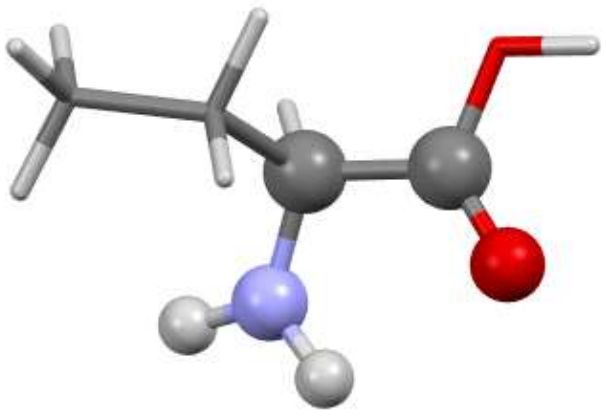 C-C-N-H = -163, 20 deg	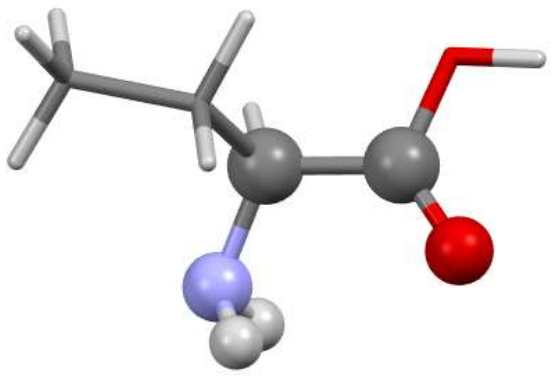 C-C-N-H = -61, 56 deg	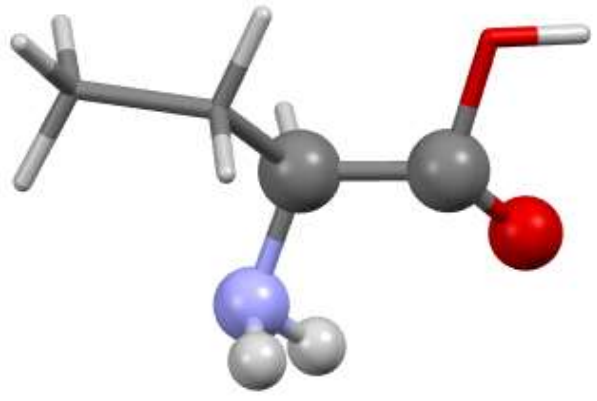 C-C-N-H = -44, 69 deg
BABA	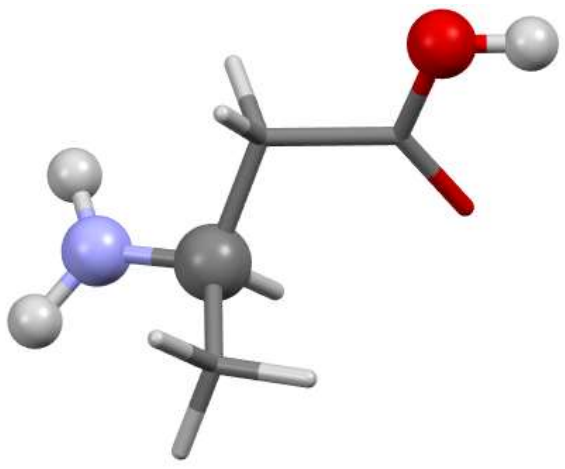	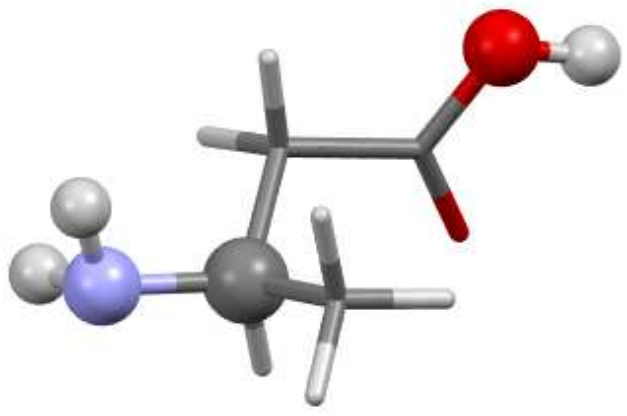	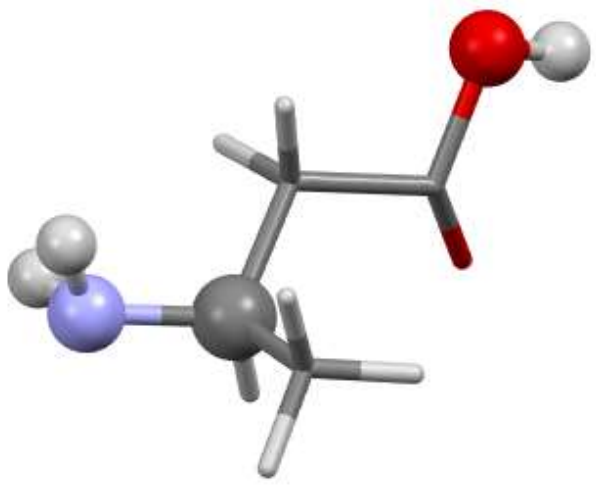
AAIBA	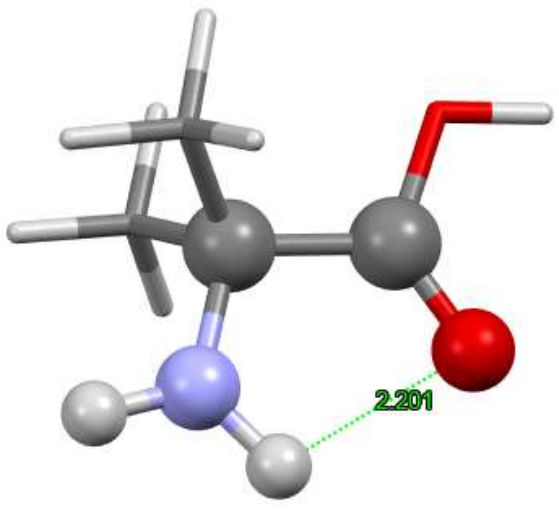 C-C-N-H = -3, 178 deg<br>O…H = 2.201	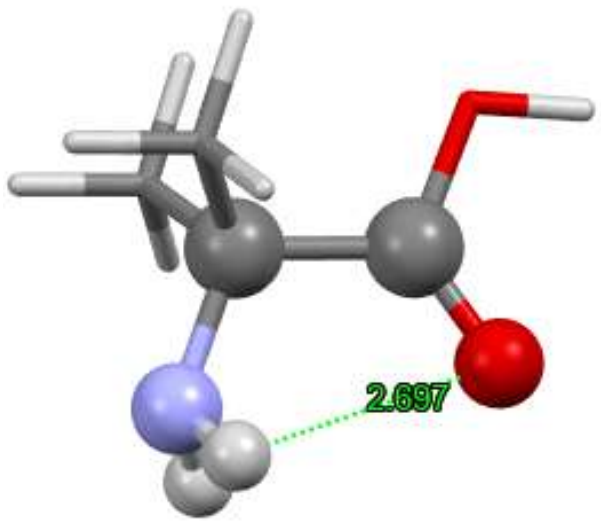 C-C-N-H = -59, 58 deg<br>O…H = 2.697	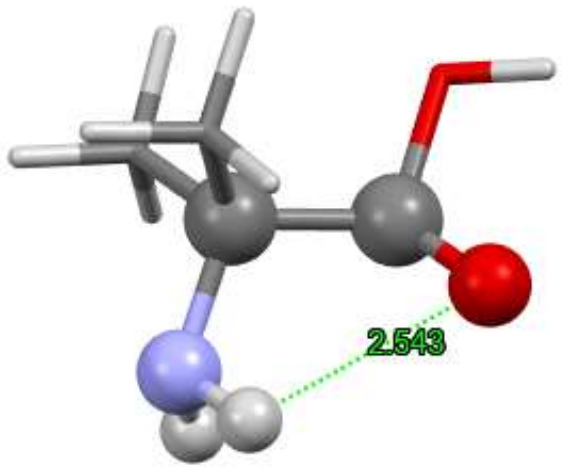 C-C-N-H = -61, 52<br>O…H = 2.543
BAIBA	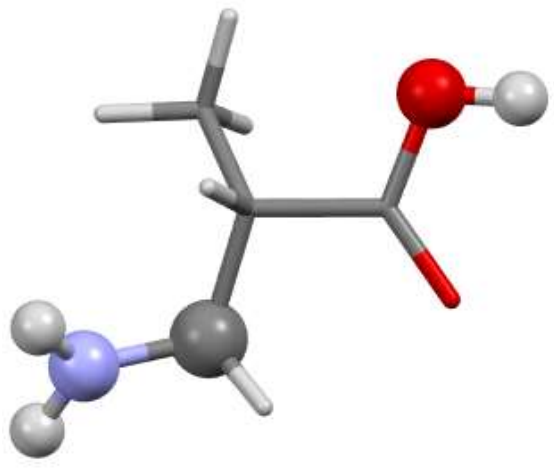	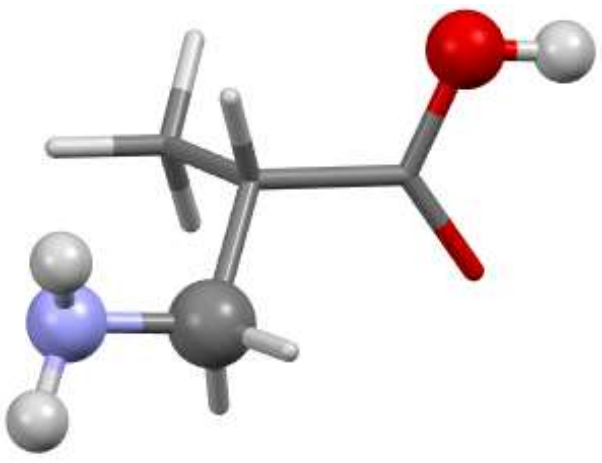	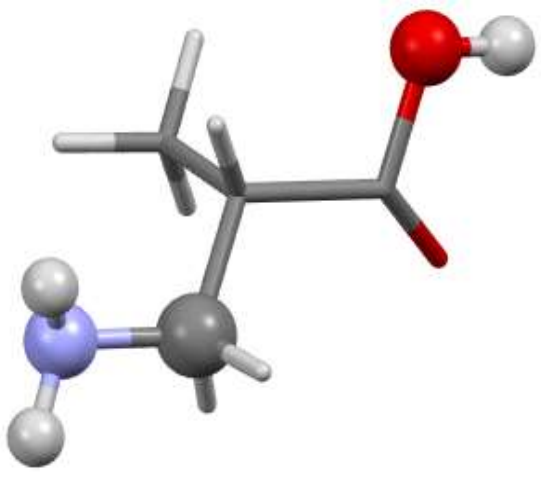

Table S5 Optimized geometries of amino acids (zwitterionic forms) in water by B3LYP/def2-TZVPD.

Z-form	L^+	L^0	L^-
AABA	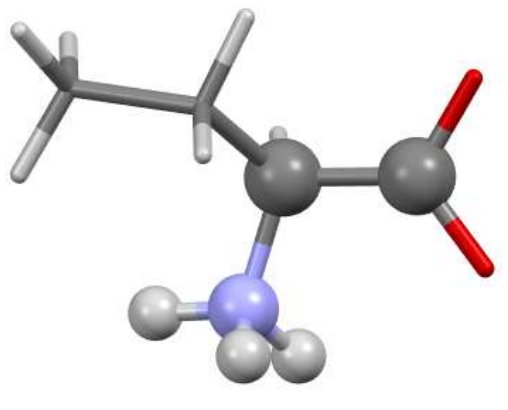<br>C-C-N-H = -174, -55, 66 deg	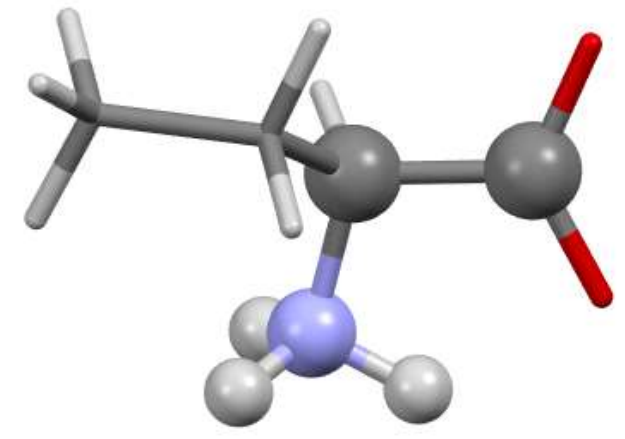<br>C-C-N-H = -117, 2, 120 deg	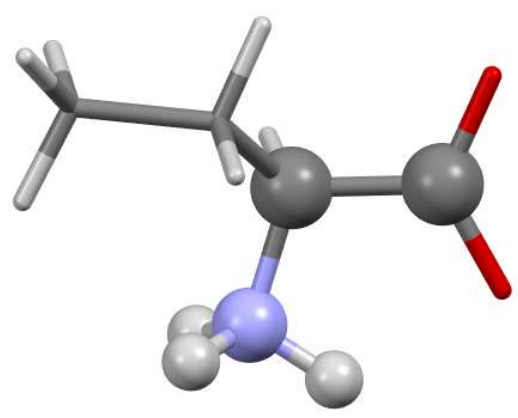<br>C-C-N-H = -120, -2, 118 deg
BABA	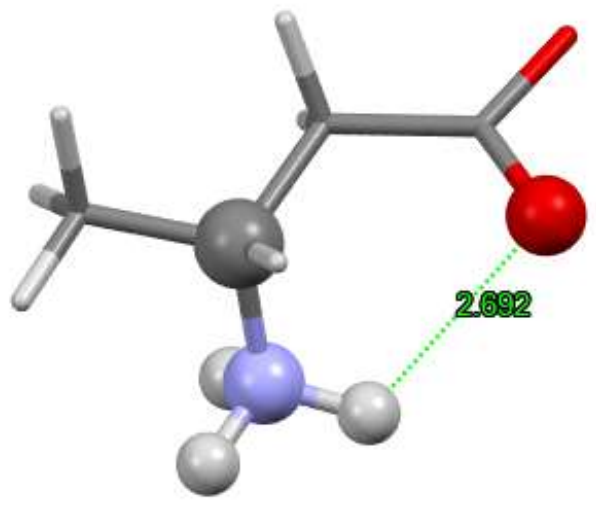<br>O…H = 2.692	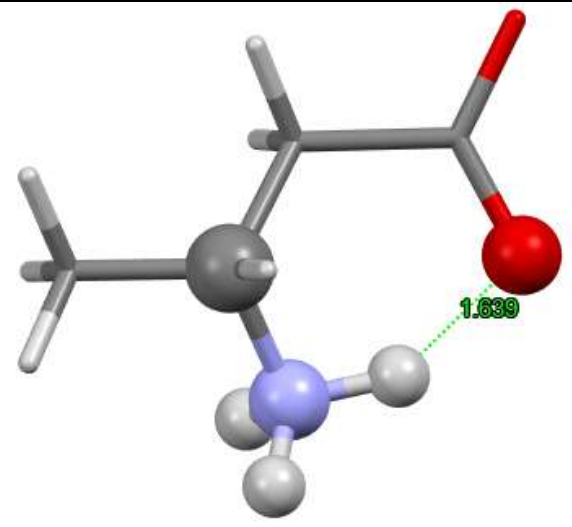<br>O…H = 1.639	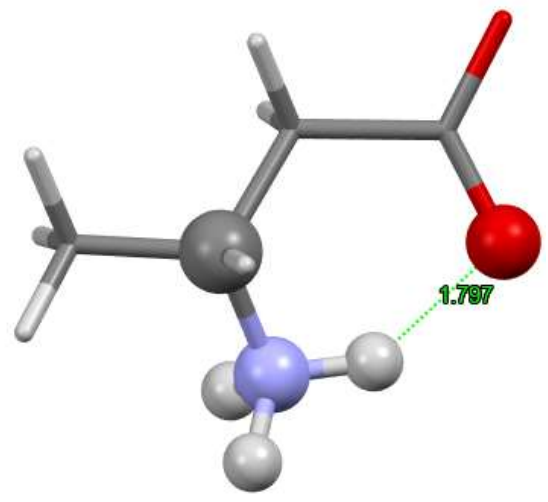<br>O…H = 1.797
AAIBA	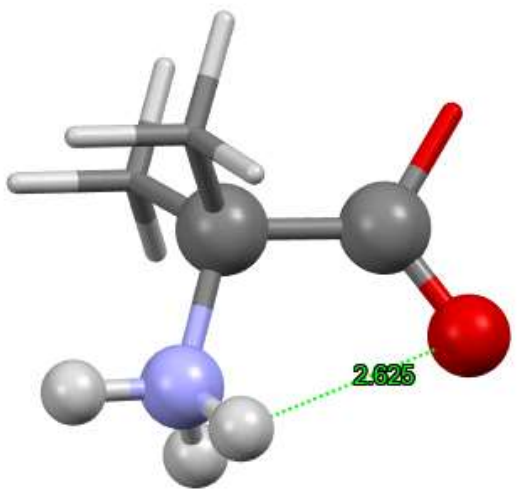<br>C-C-N-H = -64, 58, 177 deg<br>O…H = 2.626	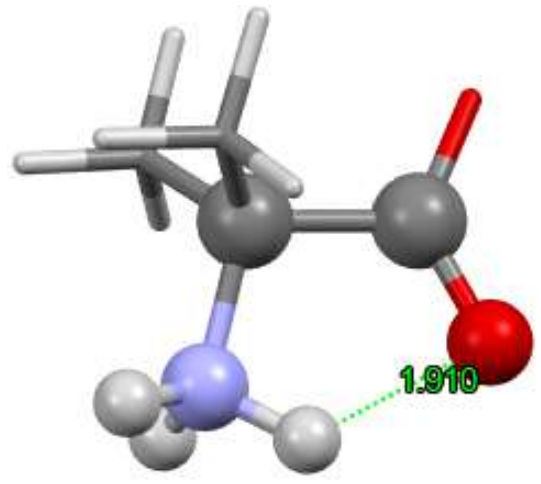<br>C-C-N-H =-93, 23, 144 deg<br>O…H = 1.910	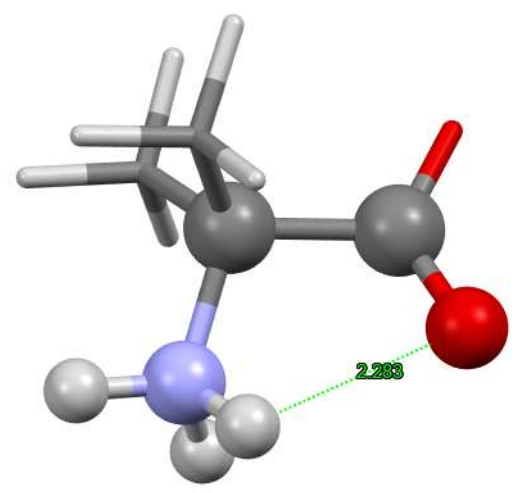<br>C-C-N-H = -65, 51, 173<br>O…H = 2.283
BAIBA	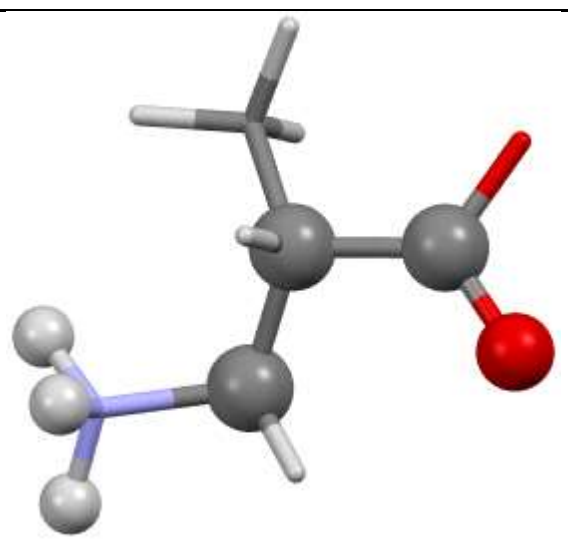	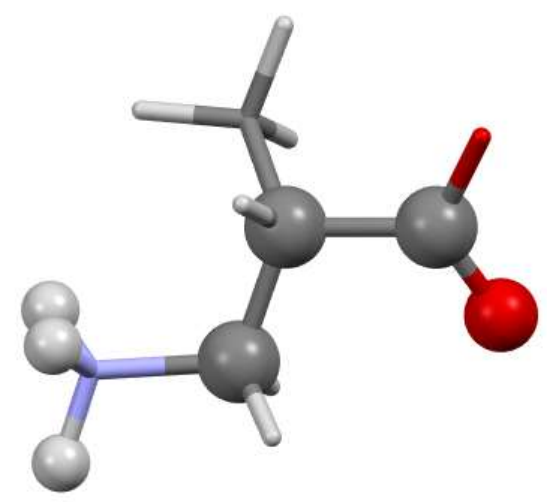	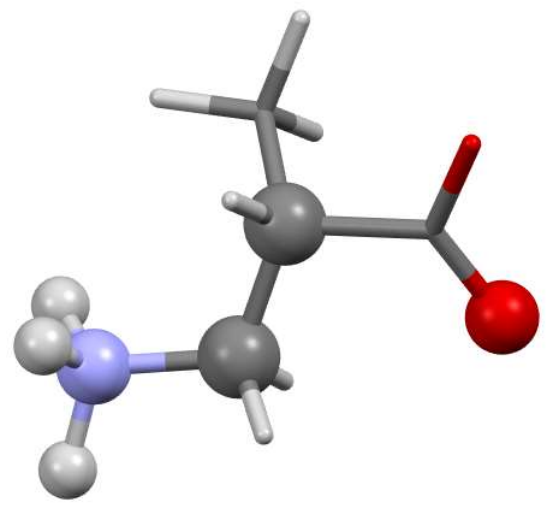

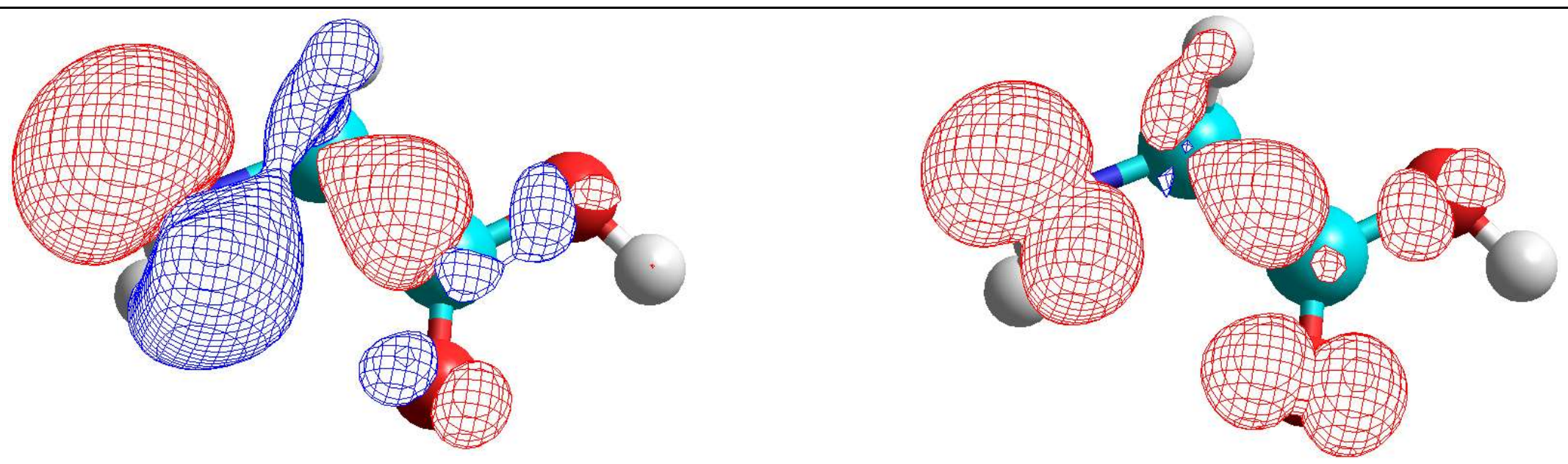

HOMO for glycine(0),
mostly localized at the nitrogen atom

Spin density for glycine(1+)

Figure S1. HOMO for glycine and spin density for glycine(1+).

Table S6. Molecular descriptors calculated by DFT-B3LYP method using adiabatic ionization/affinity processes in water for aliphatic amino acids.[a]

Characterization		Adiabatic redox properties /kcal mol^{-1}										Properties of neutral molecules						
No	Molecule	E_i	E_{eg}	χ	η	ω	$E_{ox}^{ø}$	$E_{red}^{ø}$	p	Q	α	S	V	E_{zpe}	$TS^{ø}$	HO-MO	LU-MO	ν_0
								Canonical forms, A[1]										
1A	AABA	142.3	-30.4	86.4	56.0	66.6	-6.13	1.45	1.927	-32.5	90.93	543	900	84.9	26.04	-161	-4.3	62
2A	BABA	138.4	-29.4	83.9	54.5	64.6	-5.92	1.42	3.116	-33.0	92.47	548	902	85.1	25.81	-157	-6.8	43
3A	AAIBA	142.4	-30.1	86.3	56.1	66.3	-6.11	1.43	2.076	-32.5	91.23	529	890	84.3	25.66	-163	-3.7	35
4A	BAIBA	138.1	-28.5	83.3	54.8	63.3	-5.92	1.37	2.457	-31.5	90.81	542	896	84.9	26.21	-156	-4.5	36
5A	Glycine	147.1	-25.1	86.1	61.0	60.8	-6.29	1.21	1.754	-22.7	56.8	401	623	49.6	22.08	-163	-3.4	76
6A	β-alanine	138.8	-29.7	84.3	54.6	65.1	-5.98	1.39	1.930	-26.6	73.9	477	763	67.2	24.75	-157	-3.7	8.7
7A	GABA	137.5	-29.1	83.3	54.2	64.0	-5.92	1.38	1.698	-33.9	90.9	553	903	85.1	26.31	-155	-3.0	37
8A	DAVA	135.9	-28.9	82.4	53.5	63.5	-5.83	1.36	1.821	-36.9	107.8	629	1040	102.7	28.61	-154	-2.9	15
9A	α-alanine	142.7	-29.9	86.3	56.4	66.0	-6.12	1.43	3.527	-26.7	74.3	470	760	67.1	23.99	-160	-5.4	49
10A	Valine	139.6	-29.6	84.6	55.0	65.1	-5.98	1.42	3.811	-36.7	108.2	597	1029	102.4	27.51	-159	-6.1	50
11A	Leucine	141.2	-27.4	84.3	56.9	62.4	-6.06	1.32	2.951	-42.4	124.8	667	1167	120.3	29.48	-159	-6.8	37
12A	Isoleucine	140.5	-29.5	85.0	55.5	65.1	-6.02	1.43	1.950	-42.5	124.6	676	1176	120.3	29.62	-161	-4.6	54

Table S6 (Continued)

Characterization		Adiabatic redox properties /kcal mol^{-1}							Properties of neutral molecules									
No	Molecule	E_i	E_{eg}	χ	η	ω	$E_{ox}^{ø}$	$E_{red}^{ø}$	p	Q	α	S	V	E_{zpe}	$TS^{ø}$	HO-MO	LU-MO	ν_0
								Zwitterionic forms, Z										
1Z	AABA	148.6	-18.3	83.5	65.1	53.5	-6.42	1.00	13.62	-34.0	91.89	533	893	86.0	25.69	-158	1.1	41
2Z	BABA	144.3	-16.0	80.1	64.1	50.1	-6.22	0.90	15.13	-34.1	92.53	528	882	85.9	25.07	-155	1.8	62
3Z	AAIBA	147.5	-16.7	82.1	65.4	51.5	-6.38	0.91	13.62	-32.6	91.95	529	899	85.3	25.53	-157	0.4	70
4Z	BAIBA	138.3	-19.9	79.1	59.2	52.8	-5.94	1.12	21.45	-30.2	93.30	540	897	86.7	25.75	-148	-1.9	60
5Z	Glycine	149.6	-18.9	84.3	65.4	54.3	-6.45	1.05	13.88	-21.2	58.8	393	628	50.7	21.82	-159	-0.03	77
6Z	β-alanine	139.0	-18.8	78.9	60.1	51.8	-6.00	1.11	21.69	-24.6	76.6	479	766	69.0	24.15	-148	-1.6	34
7Z	GABA	135.0	-11.9	73.5	61.6	43.8	-5.82	0.52	27.36	-28.5	93.8	554	905	86.8	26.20	-144	-1.1	43
8Z	DAVA	133.3	-11.4	72.4	61.0	42.9	-5.74	0.52	33.90	-32.0	110.8	627	1040	104.6	28.13	-143	-1.0	50
9Z	α-alanine	148.5	-18.8	83.7	64.9	54.0	-6.41	1.01	13.68	-27.1	74.9	466	769	68.2	23.61	-158	0.27	69
10Z	Valine	148.0	-18.4	83.2	64.8	53.4	-6.40	1.00	13.74	-38.6	109.7	592	1019	103.6	27.44	-157	1.41	52
11Z	Leucine	148.7	-18.5	83.6	65.1	53.7	-6.41	0.99	13.56	-47.2	126.0	665	1167	121.6	28.90	-158	0.15	52
12Z	Isoleucine	148.5	-18.9	83.7	64.8	54.1	-6.40	1.02	13.53	-44.6	125.7	667	1169	121.5	29.08	-158	0.13	62
Abbreviation		I	A	X	H	O	Eo	Er	p	Q	al	S	V	Z	ST	Ho	Lu	n

[a] Energy quantities are in kcal mol^{-1}; conversion factors: 1 kcal mol^{-1} = 4.184 kJ mol^{-1}, 1 hartree = 627.5095 kcal mol^{-1}; 1 eV = 23.06054 kcal mol^{-1}. Standard temperature $T^{ø}$ = 298.15 K. Absolute oxidation potential $E_{ox}^{ø}$ and absolute reduction potential $E_{red}^{ø}$ in V. Dipole moment p / D (*debye*, D = 3.336 × 10^{-30} A m s); isotropic quadrupole moment Q / ea_0^2, isotropic dipole polarizability α / a_0^3, solvated surface area S / a_0^2, solvated volume V / a_0^3 (*bohr*, a_0 = 5.292 ×10^{-11} m); zero-point energy E_{zpe}, lowest vibrational frequency ν_0 / cm^{-1}, total entropic term $S^{ø}T^{ø}$ in kcal mol^{-1}.

Adiabatic ionization energy $E_i = E^+ - E^0$, electron affinity $E_{eg} = E^- - E^0$, (Mulliken's) electronegativity $\chi = (E_i - E_{eg})/2$, chemical (Pearson's) hardness $\eta = (E_i + E_{eg})/2$ and (Parr's) electrophilicity index $\omega = \chi^2/2\eta$, the absolute redox potential $E_{abs}^{ø}(L^0/L^q)$ [V] = $-\Delta_{react}G^{ø}$[J mol^{-1}]/F, Faraday constant F = 96485 A s mol^{-1}.

Table S7. Molecular descriptors calculated by DLPNO-CCSD(T) method using adiabatic ionization/affinity processes in water. [a]

aug-cc-pVTZ		Adiabatic redox properties /kcal mol^{-1}							Properties of neutral molecules					
	Molecule	E_i	E_{eg}	χ	η	ω	E_{ox}^*	E_{red}^*	p	Q	S	V	HOMO	LUMO
						Canonical forms, A[1]								
1A	AABA	148.6	-27.8	88.2	60.4	64.4	-6.44	1.21	2.012	-32.3	543	900	-255	20.4
2A	BABA	144.4	-26.3	85.3	59.0	61.7	-6.26	1.14	3.338	-32.9	548	902	-249	20.4
3A	AAIBA	147.9	-27.9	87.9	60.0	64.4	-6.41	1.20	2.159	-32.4	529	891	-257	20.3
4A	BAIBA	143.9	-26.0	84.9	58.9	61.2	-6.24	1.12	2.628	-31.4	542	896	-249	20.1
5A	Glycine	148.7	-27.3	88.0	60.7	63.8	-6.45	1.18	1.891	-22.7	400	662	-260	20.4
6A	β-alanine	143.8	-26.7	85.3	58.6	62.1	-6.24	1.16	2.124	-26.6	476	761	-251	20.3
7A	GABA	142.6	-26.1	84.4	58.3	61.1	-6.18	1.13	1.770	-33.9	553	903	-247	20.6
8A	DAVA	141.2	-25.9	83.6	57.7	60.5	-6.12	1.12	1.981	-36.8	629	1040	-246	20.5
9A	α-alanine	147.9	-27.6	87.8	60.2	64.0	-6.41	1.20	3.773	-25.6	470	760	-253	20.6
10A	Valine	147.3	-26.6	87.0	60.4	62.6	-6.39	1.15	4.061	-36.6	597	1028	-252	20.1
11A	Leucine	148.4	-24.8	86.6	61.8	60.7	-6.44	1.08	3.222	-42.2	667	1167	-250	19.8
12A	Isoleucine	148.5	-26.7	87.6	60.9	63.0	-6.44	1.16	2.040	-42.4	676	1176	-254	19.9
						Zwitterionic forms, Z								
1Z	AABA	157.2	-18.4	87.8	69.4	55.5	-6.82	0.80	14.20	-33.9	533	893	-259	20.2
2Z	BABA	152.9	-16.6	84.7	68.1	52.7	-6.63	0.72	15.68	-34.0	528	882	-255	20.5
3Z	AAIBA	156.4	-16.9	86.6	69.7	53.8	-6.78	0.73	14.18	-32.5	529	899	-255	20.2
4Z	BAIBA	147.9	-19.9	83.9	64.0	55.0	-6.41	0.86	21.9	-30.0	540	897	-247	19.0
5Z	Glycine	156.3	-18.1	87.2	69.1	55.0	-6.78	0.78	14.37	-21.1	392	625	-261	20.0
6Z	β-alanine	148.0	-18.6	83.3	64.7	53.6	-6.42	0.81	22.02	-24.5	477	763	-250	18.9
7Z	GABA	143.9	-19.9	81.9	62.0	54.1	-6.24	0.86	27.82	-28.4	554	905	-247	18.7
8Z	DAVA	142.4	-20.1	81.3	61.2	54.0	-6.18	0.87	34.35	-32.0	627	1040	-244	18.6
9Z	α-alanine	157.0	-18.7	87.9	69.2	55.8	-6.81	0.81	14.24	-27.0	466	769	-259	20.1
10Z	Valine	156.9	-18.4	87.7	69.3	55.5	-6.80	0.80	14.28	-38.5	592	1018	-156	20.2
11Z	Leucine	157.5	-18.6	88.1	69.5	55.8	-6.83	0.81	14.13	-47.3	665	1167	-257	19.7
12Z	Isoleucine	157.4	-18.7	88.1	69.4	55.9	-6.83	0.81	14.12	-44.5	667	1169	-258	19.9
Abbreviation		I	A	X	H	O	Eo	Er	p	Q	S	V	Ho	Lu

For units see Table 3.

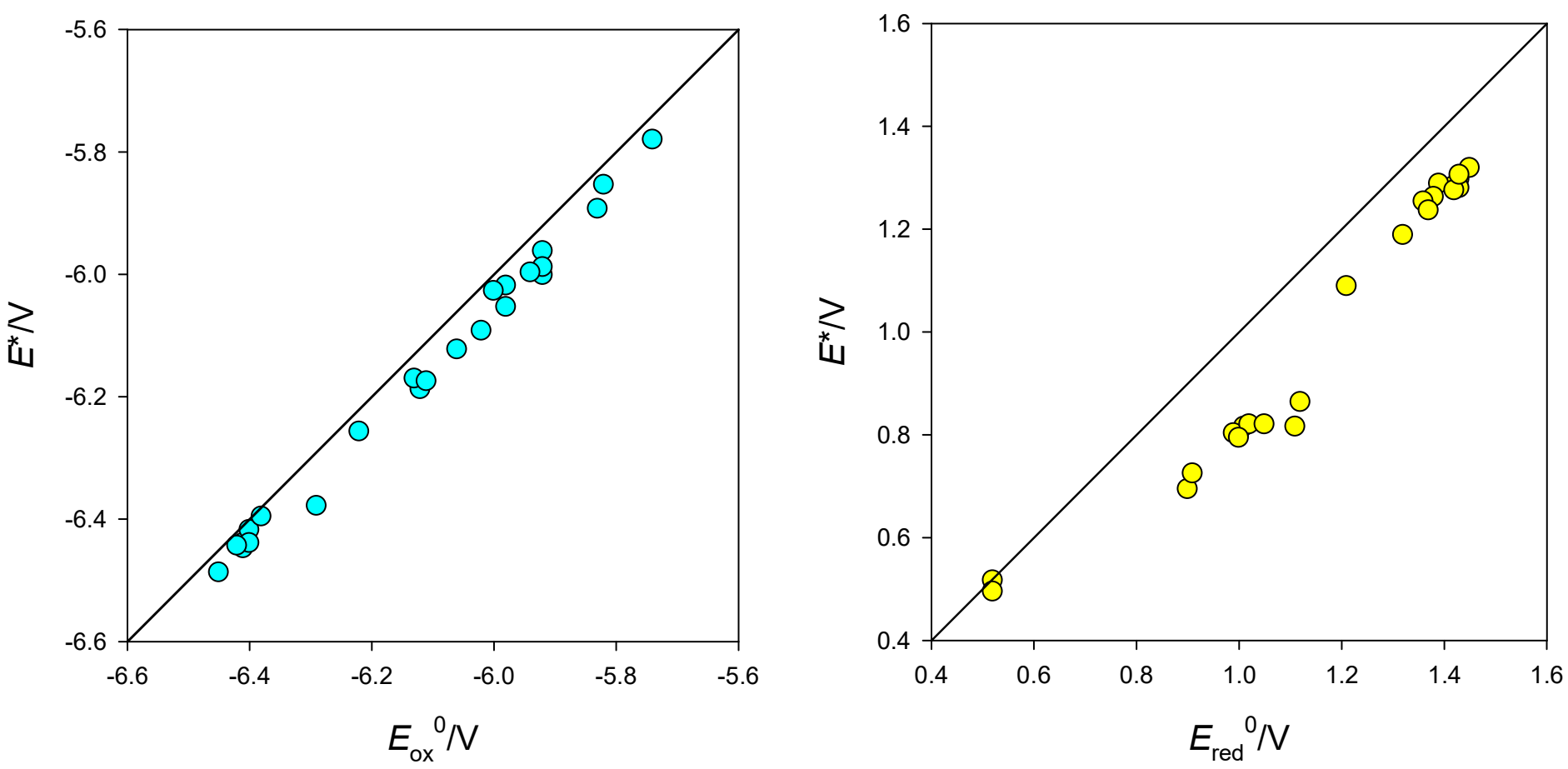

Figure S2. A relationship between absolute redox potential calculated by thermodynamic equation $E_{abs}^{o}(L^0/L^q)$ [V] = $-\Delta_{react}G^o$[J mol^{-1}]/F, and approximate formula $E_{abs}^*(L^0/L^q)$ [V] = $-\Delta_{react}E$[J mol^{-1}]/F based on ionization energy and/or electron affinity (24 items, B3LYP data). The solid line indicates a hypothetical perfect correlation.

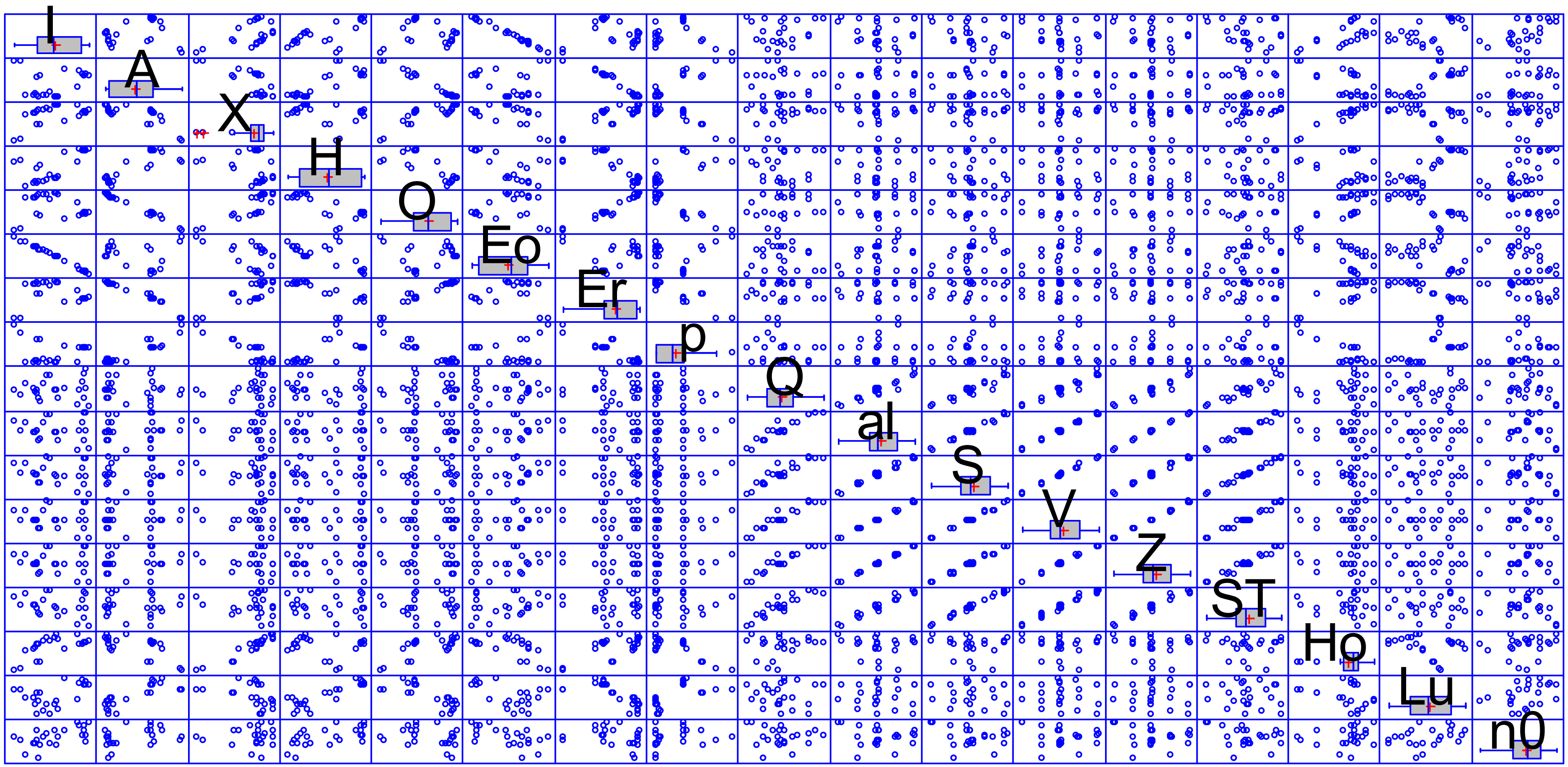

Figure S3. Matrix plot for molecular descriptors of 12 amino acids in canonical and zwitterionic forms (B3LYP data).

Quantum chemical study of molecular properties of small branched-chain amino acids in water

Roman Boča · Žofia Rádiková · Juraj Štofko · Beata Vranovičová · Cyril Rajnák

Table S8. Pair correlation coefficients between molecular descriptors of 12 amino acids in canonical and zwitterionic forms (B3LYP data).

	I	A	X	H	O	Eo	Er	p	Q	al	S	V	Z	ST	Ho	Lu	n0
I		.22	.50	.72	-.10	**-1.00**	-.09	-.08	.09	-.13	-.23	-.14	-.12	-.27	.60	.51	.60
A	.22		-.73	.84	**-.99**	-.27	**-.97**	**.92**	-.06	.05	-.01	.00	.04	-.06	-.61	.82	.39
X	.50	-.73		-.24	.81	-.45	.80	-.87	.12	-.13	-.16	-.10	-.12	-.13	**.96**	-.37	.07
H	.72	.84	-.24		-.77	-.75	-.74	.62	.00	-.04	-.14	-.08	-.04	-.20	-.10	.87	.62
O	-.10	-.99	.81	-.77		.15	**.98**	**-.95**	.08	-.06	-.02	-.02	-.05	.04	.70	-.77	-.33
Eo	-1.00	-.27	-.45	-.75	.15		.14	.02	-.09	.13	.23	.14	.12	.26	-.55	-.56	-.59
Er	-.09	-.97	.80	-.74	.98	.14		**-.91**	.06	-.08	-.04	-.04	-.07	.01	.66	-.74	-.32
p	-.08	.92	-.87	.62	-.95	.02	-.91		-.13	.07	.04	.02	.05	-.01	-.80	.64	.25
Q	.09	-.06	.12	.00	.08	-.09	.06	-.13		**.95**	**.93**	**.95**	**.95**	**.91**	.16	.00	-.12
al	-.13	.05	-.13	-.04	-.06	.13	-.08	.07	.95		**.99**	**1.00**	**1.00**	**.98**	-.09	-.03	-.17
S	-.23	-.01	-.16	-.14	-.02	.23	-.04	.04	.93	.99		**.99**	**.99**	**.99**	-.13	-.10	-.25
V	-.14	.00	-.10	-.08	-.02	.14	-.04	.02	.95	1.00	.99		**1.00**	**.99**	-.06	-.07	-.19
Z	-.12	.04	-.12	-.04	-.05	.12	-.07	.05	.95	1.00	.99	1.00		**.98**	-.07	-.03	-.18
ST	-.27	-.06	-.13	-.20	.04	.26	.01	-.01	.91	.98	.99	.99	.98		-.11	-.14	-.32
Ho	.60	-.61	.96	-.10	.70	-.55	.66	-.80	.16	-.09	-.13	-.06	-.07	-.11		-.27	.20
Lu	.51	.82	-.37	.87	-.77	-.56	-.74	.64	.00	-.03	-.10	-.07	-.03	-.14	-.27		.36
n0	.60	.39	.07	.62	-.33	-.59	-.32	.25	-.12	-.17	-.25	-.19	-.18	-.32	.20	.36	

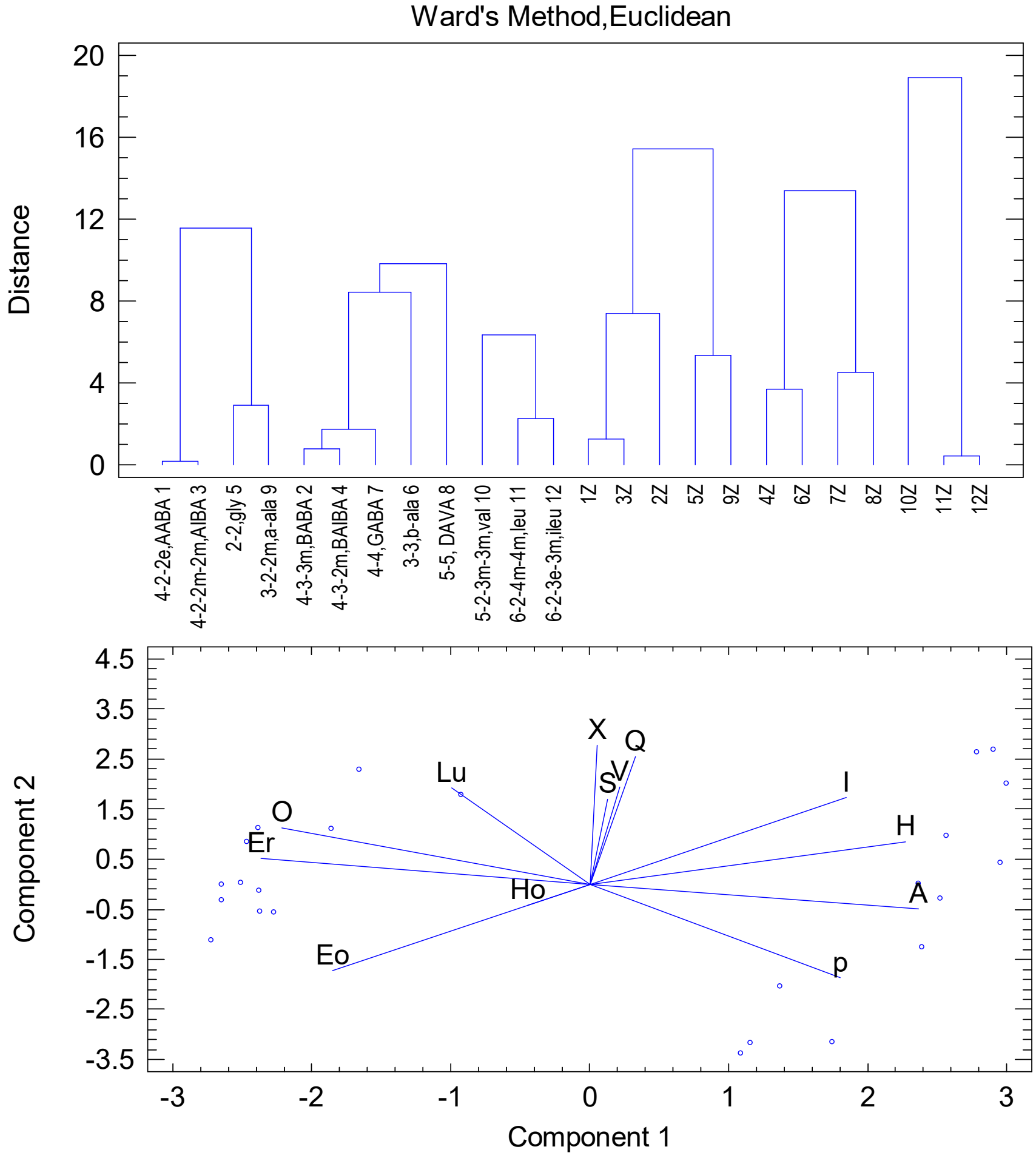

Figure S4. CA and PCA graphs for 12 amino acids in canonical and zwitterionic forms calculated by DLPNO-CCSD(T) method.
