# Supplementary material for: Quantum chemical study of molecular properties of small branched-chain amino acids in water
Source: Amino Acids. 2025 Jan 19;57(1):11. doi: 10.1007/s00726-024-03437-y (PMC11743389; doi:10.1007/s00726-024-03437-y)
Supplement: Supplementary file 1 — Supplementary file1 (PDF 1184 KB) [file 726_2024_3437_MOESM1_ESM.pdf]

Quantum chemical study of molecular properties of small branched-chain amino acids in water

Roman Boča · Žofia Rádiková · Juraj Štofko · Beata Vranovičová · Cyril Rajnák

Supplementary information

Table S1. Calculated total energy and electron correlation increments (a.u., hartree) for AABA.

Method	E^0[SCF]	E[correlation]	%	E[total]	CPU ~ M [a]
B3LYP	-363.02016906		?		M^3
Hartree-Fock	**-361.06406**723		-		M^3
MP2	**-361.06406**723	**-1.50917**625	93.7	-362.57324348	M^4, M^5
DLPNO-CCSD	**-361.06406**253	**-1.54766**712	96.1	-362.61172966	M^5
CCSD	**-361.06406**723	**-1.54757**181	96.1	-362.61163904	M^6
DLPNO-CCSD(T)	**-361.06406**723	**-1.60588**965	99.7	-362.66995688	M^6
CCSD(T)	**-361.06406**254	**-1.60997**133	100	-362.67403387	M^7

[a] AABA: 16 atoms, M = 571 basis functions (orbitals) for aug-cc-pVTZ basis set. CPU depending on basis set size M. % - percentage of the correlation energy with respect to CCSD(T). Data generated by ORCA 6.0.0. 1 hartree = 627.5095 kcal mol^{-1}.

Table S2. Structure and molecular properties for various conformers of AABA.[a]

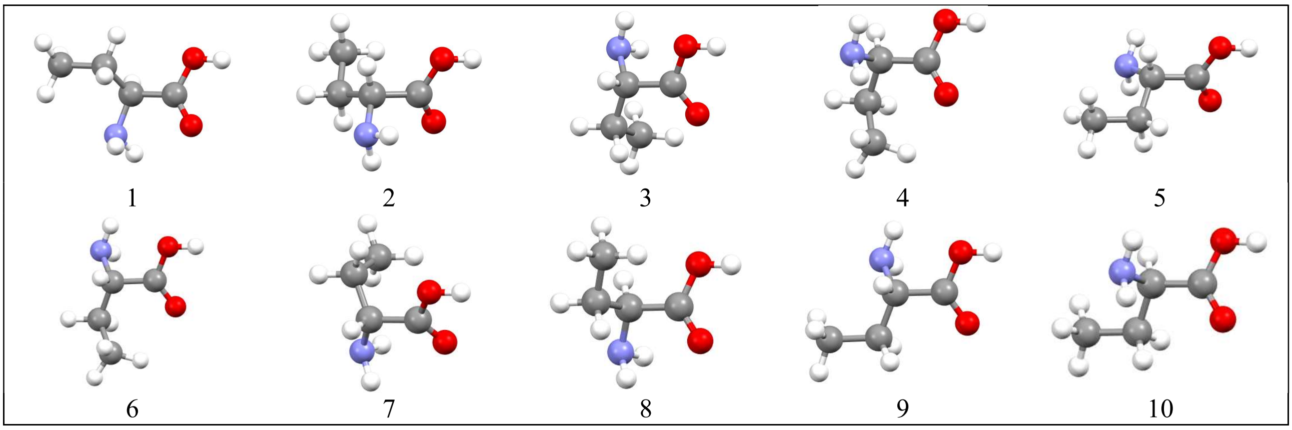

No	E^0	ΔE^0	$G^ø$	$\Delta G^ø$	p /debye	S /a_0^2	V /a_0^3	E_{zpe}	$TS^ø$	HOMO	LUMO
1	-227802.39	0	-227737.71	0	1.927	543	900	84.90	26.01	-161.14	-4.29
2	-227801.99	0.40	-227737.21	0.50	1.954	539	896	84.97	25.96	-161.69	-6.53
3	-227800.94	1.45	-227736.17	1.54	2.397	533	893	84.97	25.97	-156.81	-5.68
4	-227801.99	0.40	-227737.01	0.70	1.976	536	896	85.06	25.79	-162.57	-5.39
5	-227802.39	0.00	-227737.71	0.00	1.912	546	902	84.90	26.01	-161.04	-4.43
6	-227802.00	0.38	-227737.23	0.48	1.949	539	896	84.96	25.96	-161.62	-6.43
7	-227802.00	0.39	-227737.11	0.60	1.976	536	897	85.00	25.86	-162.58	-5.37
8	-227802.02	0.37	-227737.09	0.62	1.957	543	903	85.05	25.86	-161.71	-6.32
9	-227802.40	-0.01	-227737.66	0.05	1.920	546	902	84.93	25.97	-161.07	-4.41
10	-227802.41	-0.02	-227737.65	0.06	1.921	545	901	84.95	25.96	-161.10	-4.42

[a] All energies in kcal mol^{-1}. E^0 – total electronic energy, $G^ø$ – standard Gibbs energy, ΔE^0 and $\Delta G^ø$ energies relative to the first conformer, p – dipole moment, S – solvated surface, V – solvated volume, E_{zpe} – zero-point vibration energy, $TS^ø$ – total entropic term.

Quantum chemical study of molecular properties of small branched-chain amino acids in water

Roman Boča · Žofia Rádiková · Juraj Štofko · Beata Vranovičová · Cyril Rajnák

Table S3. Total electronic energies and standard Gibbs energies calculated by B3LYP and DLPNO-CCSD(T) methods in optimized structure of L^q.[a]

B3LYP /def2-TZVPD		Canonical forms A[1]			Zwitterionic forms Z			ΔE
Molecule/ion		A^+	A^0	A^-	Z^+	Z^0	Z^-	$\Delta G^ø$
1) AABA	E^q	-227660.07	-227802.39	-227832.78	-227658.20	**-227806.81**	-227825.15	–4.4
	$G^{ø,q}$	-227596.49	-227737.74	-227771.12	-227592.91	-227740.94	-227764.09	–3.2
2) BABA	E^q	-227665.34	-227803.78	-227833.19	-227666.32	-227810.61	-227826.61	–6.8
	$G^{ø,q}$	-227602.33	-227738.85	-227771.50	-227600.92	-227744.42	-227765.08	–5.6
3) AAIBA	E^q	-227660.77	-227803.15	-227833.30	-227660.33	-227807.82	-227824.51	–4.7
	$G^{ø,q}$	-227597.89	-227738.75	-227771.79	-227595.13	-227742.37	-227763.25	–3.6
4) BAIBA	E^q	-227662.96	-227801.11	-227829.61	-227663.81	-227802.11	-227822.04	–1.0
	$G^{ø,q}$	-227599.92	-227736.52	-227768.1	-227598.33	-227735.58	-227761.30	+0.9
		D^0	D^-					
1') AABA	E^q	-227399.6	-227510.61					
	$G^{ø,q}$	-227345.3	-227454.24					

DLPNO-CCSD(T) /aug-cc-pVTZ		Canonical forms A[1]			Zwitterionic forms Z			ΔE
Molecule/ion		A^+	A^0	A^-	Z^+	Z^0	Z^-	
1) AABA	E^q	-227430.19	-227578.79	-227606.59	-227424.70	**-227581.88**	-227600.23	–3.1
2) BABA	E^q	-227435.64	-227580.08	-227606.47	-227432.79	-227585.70	-227602.34	–5.6
3) AAIBA	E^q	-227434.12	-227582.07	-227609.96	-227428.96	-227585.35	-227602.30	–3.3
4) BAIBA	E^q	-227433.88	-227577.85	-227603.85	-227429.85	-227577.74	-227597.62	+0.11

[a] All energies in kcal mol^{-1}; conversion: 1 hartree = 627.5095 kcal mol^{-1}; 1 kcal mol^{-1} = 4.184 kJ mol^{-1}. Stability $\Delta E = E^0(Z) - E^0(A)$; $\Delta G = G^0(Z) - G^0(A)$. More stable electroneutral isomer is **bold typed**. D – deprotonated species.

Quantum chemical study of molecular properties of small branched-chain amino acids in water

Roman Boča · Žofia Rádiková · Juraj Štofko · Beata Vranovičová · Cyril Rajnák

Table S4 Optimized geometries of amino acids (canonical forms A[1]) in water by B3LYP/def2-TZVPD.

A^1 form	L^+	L^0	L^-
AABA	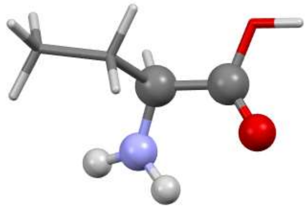 C-C-N-H = -163, 20 deg	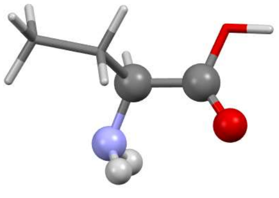 C-C-N-H = -61, 56 deg	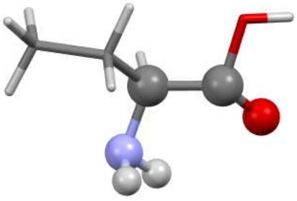 C-C-N-H = -44, 69 deg
BABA	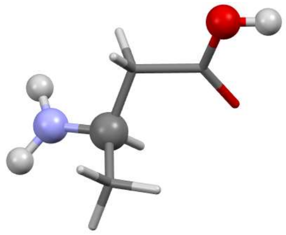	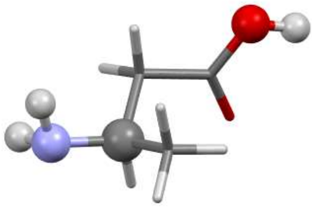	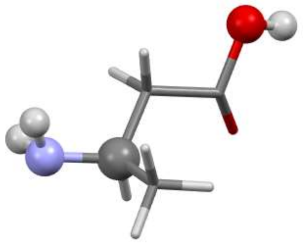
AAIBA	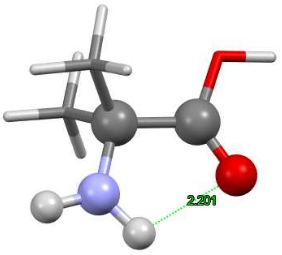 C-C-N-H = -3, 178 deg<br>O…H = 2.201	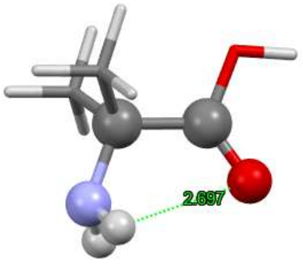 C-C-N-H = -59, 58 deg<br>O…H = 2.697	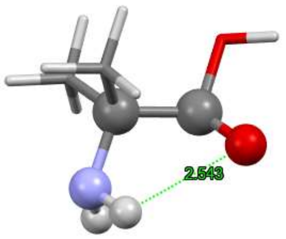 C-C-N-H = -61, 52<br>O…H = 2.543
BAIBA	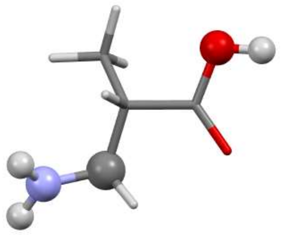	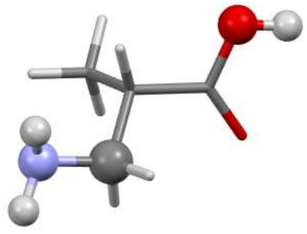	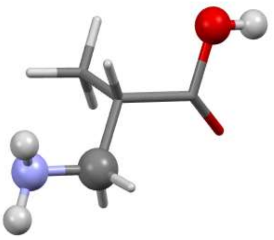

Table S5 Optimized geometries of amino acids (zwitterionic forms) in water by B3LYP/def2-TZVPD.

Z-form	L^+	L^0	L^-
AABA	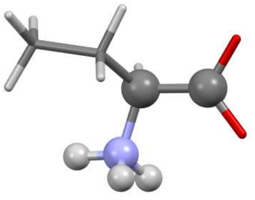 C-C-N-H = -174, -55, 66 deg	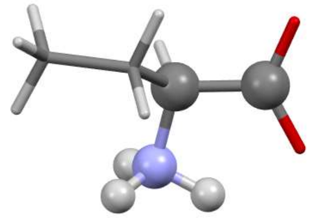 C-C-N-H = -117, 2, 120 deg	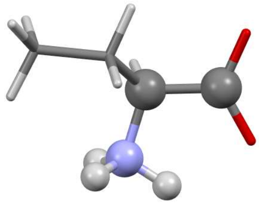 C-C-N-H = -120, -2, 118 deg
BABA	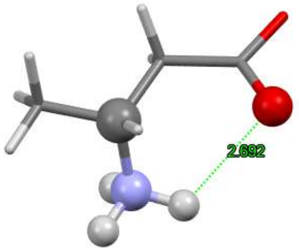 O…H = 2.692	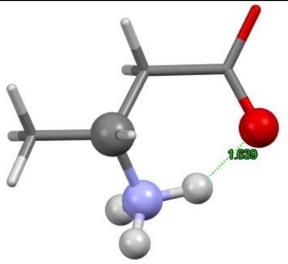 O…H = 1.639	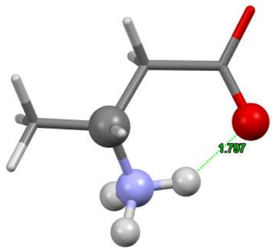 O…H = 1.797
AAIBA	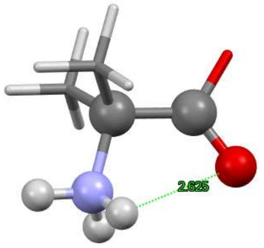 C-C-N-H = -64, 58, 177 deg O…H = 2.626	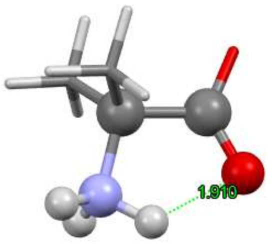 C-C-N-H =-93, 23, 144 deg O…H = 1.910	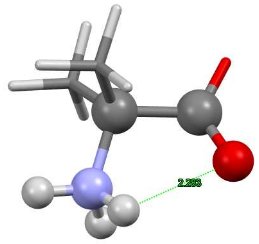 C-C-N-H = -65, 51, 173 O…H = 2.283
BAIBA	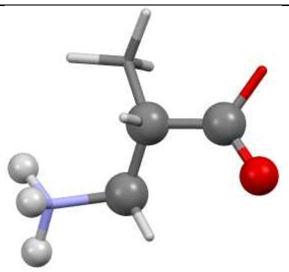	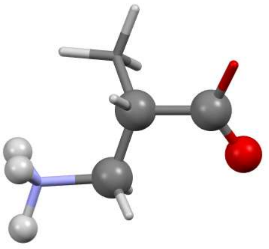	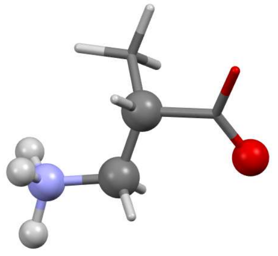

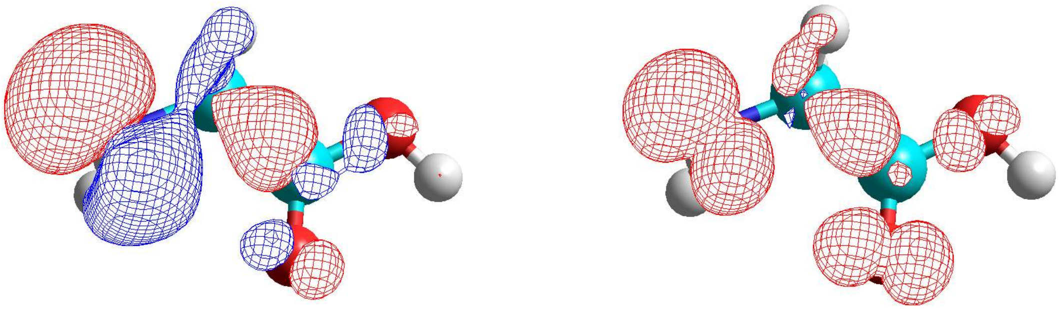

HOMO for glycine(0), mostly localized at the nitrogen atom

Spin density for glycine(1+)

Figure S1. HOMO for glycine and spin density for glycine(1+).

Table S6. Molecular descriptors calculated by DFT-B3LYP method using adiabatic ionization/affinity processes in water for aliphatic amino acids.[a]

Characterization		Adiabatic redox properties /kcal mol^{-1}										Properties of neutral molecules						
No	Molecule	E_i	E_{eg}	χ	η	ω	$E_{ox}^{\o}$	$E_{red}^{\o}$	p	Q	α	S	V	E_{zpe}	$TS^{\o}$	HO-MO	LU-MO	ν_0
								Canonical forms, A[1]										
1A	AABA	142.3	-30.4	86.4	56.0	66.6	-6.13	1.45	1.927	-32.5	90.93	543	900	84.9	26.04	-161	-4.3	62
2A	BABA	138.4	-29.4	83.9	54.5	64.6	-5.92	1.42	3.116	-33.0	92.47	548	902	85.1	25.81	-157	-6.8	43
3A	AAIBA	142.4	-30.1	86.3	56.1	66.3	-6.11	1.43	2.076	-32.5	91.23	529	890	84.3	25.66	-163	-3.7	35
4A	BAIBA	138.1	-28.5	83.3	54.8	63.3	-5.92	1.37	2.457	-31.5	90.81	542	896	84.9	26.21	-156	-4.5	36
5A	Glycine	147.1	-25.1	86.1	61.0	60.8	-6.29	1.21	1.754	-22.7	56.8	401	623	49.6	22.08	-163	-3.4	76
6A	β-alanine	138.8	-29.7	84.3	54.6	65.1	-5.98	1.39	1.930	-26.6	73.9	477	763	67.2	24.75	-157	-3.7	8.7
7A	GABA	137.5	-29.1	83.3	54.2	64.0	-5.92	1.38	1.698	-33.9	90.9	553	903	85.1	26.31	-155	-3.0	37
8A	DAVA	135.9	-28.9	82.4	53.5	63.5	-5.83	1.36	1.821	-36.9	107.8	629	1040	102.7	28.61	-154	-2.9	15
9A	α-alanine	142.7	-29.9	86.3	56.4	66.0	-6.12	1.43	3.527	-26.7	74.3	470	760	67.1	23.99	-160	-5.4	49
10A	Valine	139.6	-29.6	84.6	55.0	65.1	-5.98	1.42	3.811	-36.7	108.2	597	1029	102.4	27.51	-159	-6.1	50
11A	Leucine	141.2	-27.4	84.3	56.9	62.4	-6.06	1.32	2.951	-42.4	124.8	667	1167	120.3	29.48	-159	-6.8	37
12A	Isoleucine	140.5	-29.5	85.0	55.5	65.1	-6.02	1.43	1.950	-42.5	124.6	676	1176	120.3	29.62	-161	-4.6	54

Table S6 (Continued)

Characterization		Adiabatic redox properties /kcal mol^{-1}										Properties of neutral molecules					
No	Molecule	E_i	E_{eg}	χ	η	ω	$E_{ox}^{ø}$	$E_{red}^{ø}$	p	Q	α	S	V	E_{zpe}	$TS^{ø}$	HO-MO	LU-MO	ν_0
								Zwitterionic forms, Z										
1Z	AABA	148.6	-18.3	83.5	65.1	53.5	-6.42	1.00	13.62	-34.0	91.89	533	893	86.0	25.69	-158	1.1	41
2Z	BABA	144.3	-16.0	80.1	64.1	50.1	-6.22	0.90	15.13	-34.1	92.53	528	882	85.9	25.07	-155	1.8	62
3Z	AAIBA	147.5	-16.7	82.1	65.4	51.5	-6.38	0.91	13.62	-32.6	91.95	529	899	85.3	25.53	-157	0.4	70
4Z	BAIBA	138.3	-19.9	79.1	59.2	52.8	-5.94	1.12	21.45	-30.2	93.30	540	897	86.7	25.75	-148	-1.9	60
5Z	Glycine	149.6	-18.9	84.3	65.4	54.3	-6.45	1.05	13.88	-21.2	58.8	393	628	50.7	21.82	-159	-0.03	77
6Z	β-alanine	139.0	-18.8	78.9	60.1	51.8	-6.00	1.11	21.69	-24.6	76.6	479	766	69.0	24.15	-148	-1.6	34
7Z	GABA	135.0	-11.9	73.5	61.6	43.8	-5.82	0.52	27.36	-28.5	93.8	554	905	86.8	26.20	-144	-1.1	43
8Z	DAVA	133.3	-11.4	72.4	61.0	42.9	-5.74	0.52	33.90	-32.0	110.8	627	1040	104.6	28.13	-143	-1.0	50
9Z	α-alanine	148.5	-18.8	83.7	64.9	54.0	-6.41	1.01	13.68	-27.1	74.9	466	769	68.2	23.61	-158	0.27	69
10Z	Valine	148.0	-18.4	83.2	64.8	53.4	-6.40	1.00	13.74	-38.6	109.7	592	1019	103.6	27.44	-157	1.41	52
11Z	Leucine	148.7	-18.5	83.6	65.1	53.7	-6.41	0.99	13.56	-47.2	126.0	665	1167	121.6	28.90	-158	0.15	52
12Z	Isoleucine	148.5	-18.9	83.7	64.8	54.1	-6.40	1.02	13.53	-44.6	125.7	667	1169	121.5	29.08	-158	0.13	62
Abbreviation		I	A	X	H	O	Eo	Er	p	Q	al	S	V	Z	ST	Ho	Lu	n

[a] Energy quantities are in kcal mol^{-1}; conversion factors: 1 kcal mol^{-1} = 4.184 kJ mol^{-1}, 1 hartree = 627.5095 kcal mol^{-1}; 1 eV = 23.06054 kcal mol^{-1}. Standard temperature $T^{ø}$ = 298.15 K. Absolute oxidation potential $E_{ox}^{ø}$ and absolute reduction potential $E_{red}^{ø}$ in V. Dipole moment p / D (*debye*, D = 3.336 × 10^{-30} A m s); isotropic quadrupole moment Q / ea_0^2, isotropic dipole polarizability α / a_0^3, solvated surface area S / a_0^2, solvated volume V / a_0^3 (*bohr*, a_0 = 5.292 ×10^{-11} m); zero-point energy E_{zpe}, lowest vibrational frequency ν_0 / cm^{-1}, total entropic term $S^{ø}T^{ø}$ in kcal mol^{-1}.

Adiabatic ionization energy $E_i = E^+ - E^0$, electron affinity $E_{eg} = E^- - E^0$, (Mulliken's) electronegativity $\chi = (E_i - E_{eg})/2$, chemical (Pearson's) hardness $\eta = (E_i + E_{eg})/2$ and (Parr's) electrophilicity index $\omega = \chi^2/2\eta$, the absolute redox potential $E_{abs}^{ø}(L^0/L^q)$ [V] = $-\Delta_{react}G^{ø}$[J mol^{-1}]/F, Faraday constant F = 96485 A s mol^{-1}.

Table S7. Molecular descriptors calculated by DLPNO-CCSD(T) method using adiabatic ionization/affinity processes in water. [a]

aug-cc-pVTZ		Adiabatic redox properties /kcal mol^{-1}								Properties of neutral molecules				
	Molecule	E_i	E_{eg}	χ	η	ω	E_{ox}^*	E_{red}^*	p	Q	S	V	HOMO	LUMO
						Canonical forms, A[1]								
1A	AABA	148.6	-27.8	88.2	60.4	64.4	-6.44	1.21	2.012	-32.3	543	900	-255	20.4
2A	BABA	144.4	-26.3	85.3	59.0	61.7	-6.26	1.14	3.338	-32.9	548	902	-249	20.4
3A	AAIBA	147.9	-27.9	87.9	60.0	64.4	-6.41	1.20	2.159	-32.4	529	891	-257	20.3
4A	BAIBA	143.9	-26.0	84.9	58.9	61.2	-6.24	1.12	2.628	-31.4	542	896	-249	20.1
5A	Glycine	148.7	-27.3	88.0	60.7	63.8	-6.45	1.18	1.891	-22.7	400	662	-260	20.4
6A	β-alanine	143.8	-26.7	85.3	58.6	62.1	-6.24	1.16	2.124	-26.6	476	761	-251	20.3
7A	GABA	142.6	-26.1	84.4	58.3	61.1	-6.18	1.13	1.770	-33.9	553	903	-247	20.6
8A	DAVA	141.2	-25.9	83.6	57.7	60.5	-6.12	1.12	1.981	-36.8	629	1040	-246	20.5
9A	α-alanine	147.9	-27.6	87.8	60.2	64.0	-6.41	1.20	3.773	-25.6	470	760	-253	20.6
10A	Valine	147.3	-26.6	87.0	60.4	62.6	-6.39	1.15	4.061	-36.6	597	1028	-252	20.1
11A	Leucine	148.4	-24.8	86.6	61.8	60.7	-6.44	1.08	3.222	-42.2	667	1167	-250	19.8
12A	Isoleucine	148.5	-26.7	87.6	60.9	63.0	-6.44	1.16	2.040	-42.4	676	1176	-254	19.9
						Zwitterionic forms, Z								
1Z	AABA	157.2	-18.4	87.8	69.4	55.5	-6.82	0.80	14.20	-33.9	533	893	-259	20.2
2Z	BABA	152.9	-16.6	84.7	68.1	52.7	-6.63	0.72	15.68	-34.0	528	882	-255	20.5
3Z	AAIBA	156.4	-16.9	86.6	69.7	53.8	-6.78	0.73	14.18	-32.5	529	899	-255	20.2
4Z	BAIBA	147.9	-19.9	83.9	64.0	55.0	-6.41	0.86	21.9	-30.0	540	897	-247	19.0
5Z	Glycine	156.3	-18.1	87.2	69.1	55.0	-6.78	0.78	14.37	-21.1	392	625	-261	20.0
6Z	β-alanine	148.0	-18.6	83.3	64.7	53.6	-6.42	0.81	22.02	-24.5	477	763	-250	18.9
7Z	GABA	143.9	-19.9	81.9	62.0	54.1	-6.24	0.86	27.82	-28.4	554	905	-247	18.7
8Z	DAVA	142.4	-20.1	81.3	61.2	54.0	-6.18	0.87	34.35	-32.0	627	1040	-244	18.6
9Z	α-alanine	157.0	-18.7	87.9	69.2	55.8	-6.81	0.81	14.24	-27.0	466	769	-259	20.1
10Z	Valine	156.9	-18.4	87.7	69.3	55.5	-6.80	0.80	14.28	-38.5	592	1018	-156	20.2
11Z	Leucine	157.5	-18.6	88.1	69.5	55.8	-6.83	0.81	14.13	-47.3	665	1167	-257	19.7
12Z	Isoleucine	157.4	-18.7	88.1	69.4	55.9	-6.83	0.81	14.12	-44.5	667	1169	-258	19.9
Abbreviation		I	A	X	H	O	Eo	Er	p	Q	S	V	Ho	Lu

For units see Table 3.

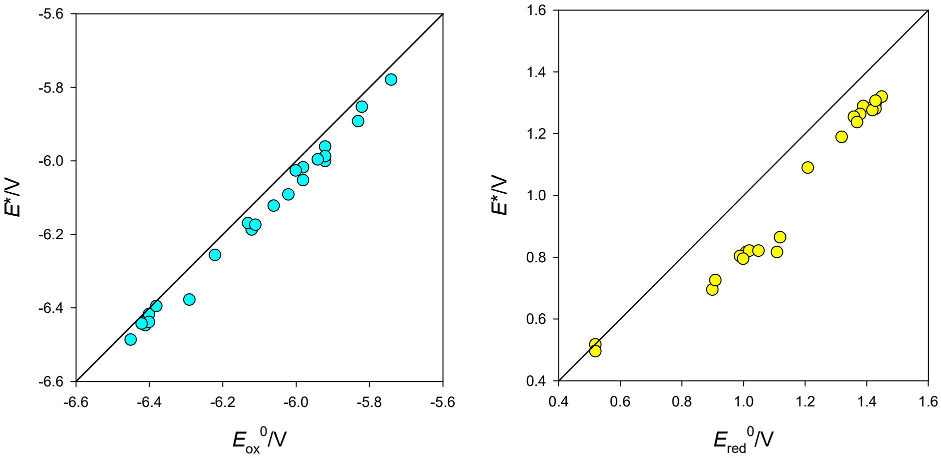

Figure S2. A relationship between absolute redox potential calculated by thermodynamic equation $E_{abs}{}^{o}(L^0/L^q)$ [V] = $-\Delta_{react}G^{o}$[J mol^{-1}]/F, and approximate formula $E_{abs}{}^{*}(L^0/L^q)$ [V] = $-\Delta_{react}E$[J mol^{-1}]/F based on ionization energy and/or electron affinity (24 items, B3LYP data). The solid line indicates a hypothetical perfect correlation.

Roman Boča · Žofia Rádiková · Juraj Štofko · Beata Vranovičová · Cyril Rajnák

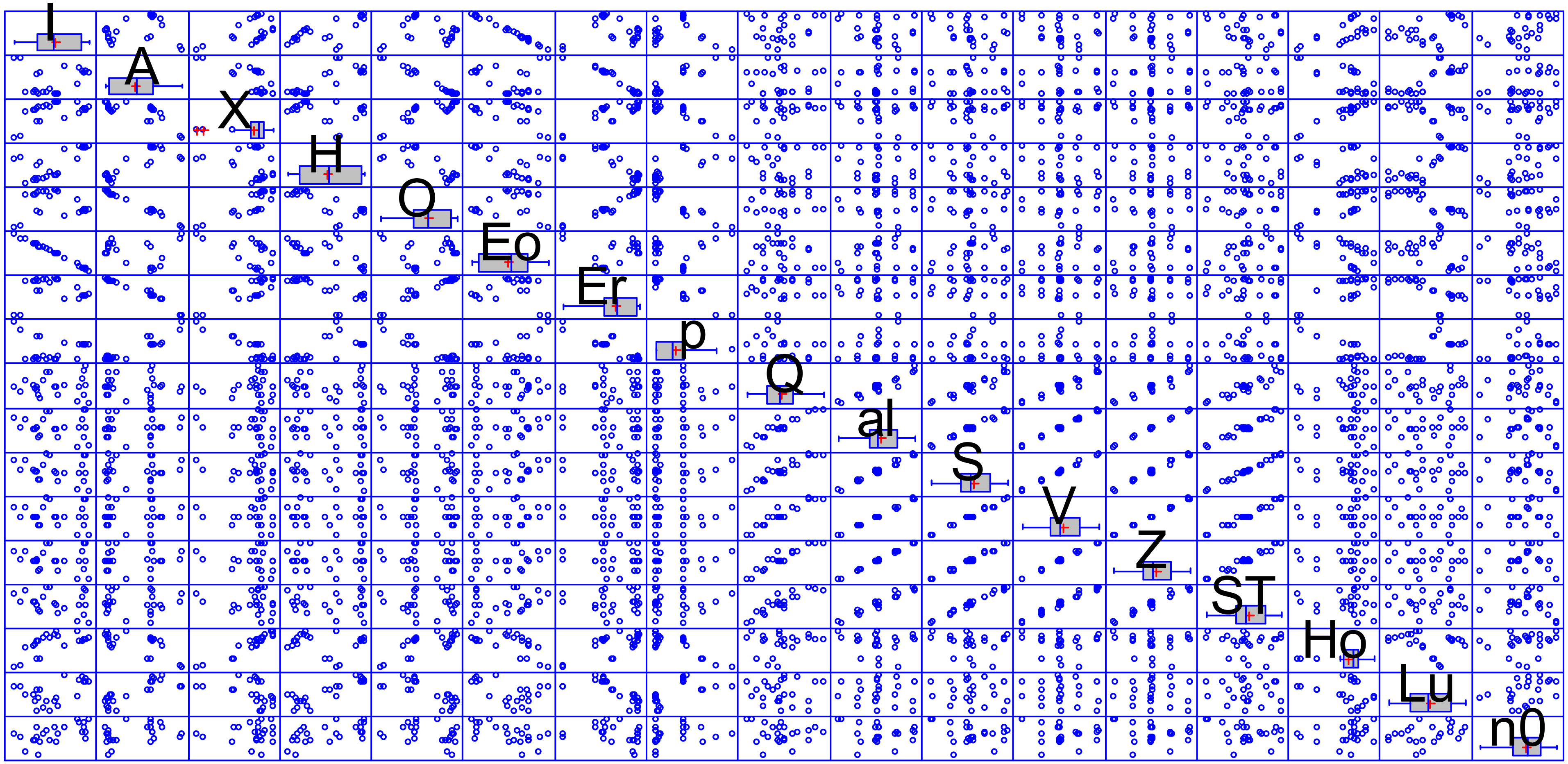

Figure S3. Matrix plot for molecular descriptors of 12 amino acids in canonical and zwitterionic forms (B3LYP data).

Table S8. Pair correlation coefficients between molecular descriptors of 12 amino acids in canonical and zwitterionic forms (B3LYP data).

	I	A	X	H	O	Eo	Er	p	Q	al	S	V	Z	ST	Ho	Lu	n0
I		.22	.50	.72	-.10	**-1.00**	-.09	-.08	.09	-.13	-.23	-.14	-.12	-.27	.60	.51	.60
A	.22		-.73	.84	**-.99**	-.27	**-.97**	**.92**	-.06	.05	-.01	.00	.04	-.06	-.61	.82	.39
X	.50	-.73		-.24	.81	-.45	.80	-.87	.12	-.13	-.16	-.10	-.12	-.13	**.96**	-.37	.07
H	.72	.84	-.24		-.77	-.75	-.74	.62	.00	-.04	-.14	-.08	-.04	-.20	-.10	.87	.62
O	-.10	-.99	.81	-.77		.15	**.98**	**-.95**	.08	-.06	-.02	-.02	-.05	.04	.70	-.77	-.33
Eo	-1.00	-.27	-.45	-.75	.15		.14	.02	-.09	.13	.23	.14	.12	.26	-.55	-.56	-.59
Er	-.09	-.97	.80	-.74	.98	.14		**-.91**	.06	-.08	-.04	-.04	-.07	.01	.66	-.74	-.32
p	-.08	.92	-.87	.62	-.95	.02	-.91		-.13	.07	.04	.02	.05	-.01	-.80	.64	.25
Q	.09	-.06	.12	.00	.08	-.09	.06	-.13		**.95**	**.93**	**.95**	**.95**	**.91**	.16	.00	-.12
al	-.13	.05	-.13	-.04	-.06	.13	-.08	.07	.95		**.99**	**1.00**	**1.00**	**.98**	-.09	-.03	-.17
S	-.23	-.01	-.16	-.14	-.02	.23	-.04	.04	.93	.99		**.99**	**.99**	**.99**	-.13	-.10	-.25
V	-.14	.00	-.10	-.08	-.02	.14	-.04	.02	.95	1.00	.99		**1.00**	**.99**	-.06	-.07	-.19
Z	-.12	.04	-.12	-.04	-.05	.12	-.07	.05	.95	1.00	.99	1.00		**.98**	-.07	-.03	-.18
ST	-.27	-.06	-.13	-.20	.04	.26	.01	-.01	.91	.98	.99	.99	.98		-.11	-.14	-.32
Ho	.60	-.61	.96	-.10	.70	-.55	.66	-.80	.16	-.09	-.13	-.06	-.07	-.11		-.27	.20
Lu	.51	.82	-.37	.87	-.77	-.56	-.74	.64	.00	-.03	-.10	-.07	-.03	-.14	-.27		.36
n0	.60	.39	.07	.62	-.33	-.59	-.32	.25	-.12	-.17	-.25	-.19	-.18	-.32	.20	.36	

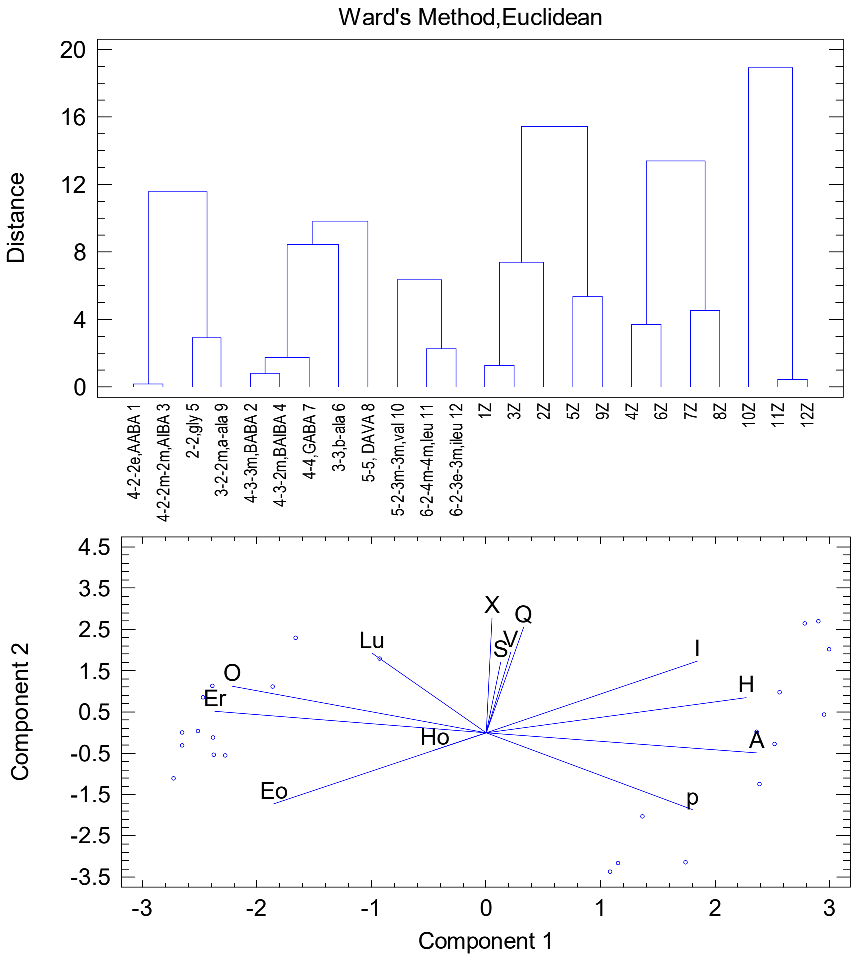

Figure S4. CA and PCA graphs for 12 amino acids in canonical and zwitterionic forms calculated by DLPNO-CCSD(T) method.
